# The Differential Expression of Mevalonate Pathway Genes in the Gut of the Bark Beetle *Dendroctonus rhizophagus* (Curculionidae: Scolytinae) Is Unrelated to the de Novo Synthesis of Terpenoid Pheromones

**DOI:** 10.3390/ijms20164011

**Published:** 2019-08-17

**Authors:** Laura Elisa Sarabia, María Fernanda López, Gabriel Obregón-Molina, Claudia Cano-Ramírez, Guillermo Sánchez-Martínez, Gerardo Zúñiga

**Affiliations:** 1 Laboratorio de Variación Biológica y Evolución, Departamento de Zoología, Escuela, Nacional de Ciencias Biológicas, Instituto Politécnico Nacional, Prolongación de Carpio y Plan de Ayala s/n, Miguel Hidalgo, Mexico City CP 11340, Mexico; 2 Instituto Nacional de Investigaciones Forestales, Agrícolas y Pecuarias, Campo Experimental Pabellón, Km. 32.5 Carr. Ags.-Zac., Pabellón de Arteaga, Ags. CP 20660, Mexico

**Keywords:** mevalonate pathway, *Dendroctonus*, juvenile hormone III, pheromone production

## Abstract

Bark beetles commonly produce de novo terpenoid pheromones using precursors synthesized through the mevalonate pathway. This process is regulated by Juvenile Hormone III (JH III). In this work, the expression levels of mevalonate pathway genes were quantified after phloem feeding—to induce the endogenous synthesis of JH III—and after the topical application of a JH III solution. The mevalonate pathway genes from *D*. *rhizophagus* were cloned, molecularly characterized, and their expression levels were quantified. Also, the terpenoid compounds produced in the gut were identified and quantified by Gas Chromatography Mass Spectrometry (GC-MS). The feeding treatment produced an evident upregulation, mainly in acetoacetyl-CoA thiolase (*AACT*), 3-hydroxy-3-methylglutaryl-CoA synthase (*HMGS*), 3-hydroxy-3-methylglutaryl-CoA reductase (*HMGR*), phosphomevalonate kinase (*PMK*), and isopentenyl diphosphate isomerase (*IPPI*) genes, and males reached higher expression levels compared to females. In contrast, the JH III treatment did not present a clear pattern of upregulation in any sex or time. Notably, the genes responsible for the synthesis of frontalin and ipsdienol precursors (geranyl diphosphate synthase/farnesyl diphosphate synthase (*GPPS*/*FPPS*) and geranylgeranyl diphosphate synthase (*GGPPS*)) were not clearly upregulated, nor were these compounds further identified. Furthermore, trans-verbenol and myrtenol were the most abundant compounds in the gut, which are derived from an α-pinene transformation rather than de novo synthesis. Hence, the expression of mevalonate pathway genes in *D*. *rhizophagus* gut is not directed to the production of terpenoid pheromones, regardless of their frequent occurrence in the genus *Dendroctonus*.

## 1. Introduction

*Dendroctonus* bark beetles (Curculionidae: Scolytinae) are key ecological agents of coniferous forests, and some of their species are the most destructive pests in these communities in North and Central America [1,2]. These bark beetles complete their life cycles under the bark of host trees, except for a brief dispersal period during which adults find a new host and mate. These insects locate and colonize their host trees through the detection of specific blends of terpenoid compounds (kairomones) produced by the trees, as well as aggregation and antiaggregation pheromones [3,4]. Aggregation pheromones are essential either as sex attractants or as coordinators of massive attacks against the hosts, whereas antiaggregation pheromones regulate the population of conspecifics that can settle on the trees [5]. 

Pheromone production in bark beetles, including the *Dendroctonus* species, is an adaptive strategy favored by natural selection because pheromones provide information about food availability and sources, conspecific attraction, reproductive partners, and regulation of population density [6]. In these insects, pheromone production has several ecological sources, such as microbial assisted synthesis [7], autoxidation of host compounds [8], sequestration and release of host compounds [9], transformation of host terpenes to pheromonal compounds [10], and endogenous production [4,11,12,13,14,15]. Pheromone production can vary based on insect health, mating system, colonization strategy, species aggressiveness, and geographical region [16,17,18,19]. 

The mevalonate (MVA) pathway is an endogenous metabolic route present in eukaryotes, archaea, and some eubacteria. This route is directly involved in the synthesis of cholesterol, hormones, and other isoprenoids metabolites [20,21,22]. Hemi- and monoisoprenoids compounds, as well as bicyclic acetals used as aggregation or antiaggregation pheromones by bark beetles, are produced through de novo synthesis in the gut of these insects [4,23,24,25,26,27,28]. Although it is not known how the MVA pathway is regulated in bark beetles, it has been demonstrated that feeding on phloem triggers the synthesis of Juvenile Hormone III (JH III) in the corpora allata [29], thereby regulating transcription of the genes coding for the enzymes that catalyze the synthesis of terpenoid backbones (*AACT*, acetoacetyl-CoA thiolase; *HMGS*, 3-hydroxy-3-methylglutaryl-CoA synthase; *HMGR*, 3-hydroxy-3-methylglutaryl-CoA reductase; *MK*, mevalonate kinase; *PMK*, phosphomevalonate kinase; *MDPC*, diphosphomevalonate decarboxylase; *IPPI*, isopentenyl diphosphate isomerase; *GPPS*, geranyl diphosphate synthase; *FPPS*, farnesyl diphosphate synthase; *GGPPS*, geranylgeranyl diphosphate synthase) [30,31]. 

Most of these enzymes synthesize a unique product, except for GPPS and FPPS, which can be mono- or bifunctional (GPPS/FPPS) [32], as reported in *Phaedon cochleariae* [33], *Manduca sexta* [34], *Myzus persicae* [35], and *Dendroctonus ponderosae* [36]. 

*Dendroctonus rhizophagus* Thomas and Bright is an endemic species of the Sierra Madre Occidental in Mexico and an atypical species within the *Dendroctonus* genus because generally just one or two pairs, rather than tens to thousands of pairs, colonize and kill a single host of seedlings or saplings <3.0 m high and <20 cm in diameter of 11 pine species (Pinales, Pinaceae), mainly *Pinus arizonica* Engelm., *P. engelmannii* Carr., *P. leiophylla* Schlecht and Cham, and *P. durangensis* Martínez [37,38]. The life cycle of this bark beetle is univoltine and synchronous. Females lay their eggs in groups, and when larvae hatch, they feed gregariously on the phloem of the tree stem. During winter, larvae remain in the roots to survive the low temperatures, and in early spring, they regroup in the primary roots to transform into pupae and adults. Females and males emerge in the early summer to colonize a new host and mate [39]. Chemical ecology studies in *D. rhizophagus* have reported high levels of trans-verbenol, as well as other oxygenated monoterpenes, such as myrtenal, myrtenol, cis-verbenol, fenchyl alcohol, and verbenone, but not frontalin or ipsdienol [40]. Whereas ipsdienol has only been identified in *D*. *mesoamericanus* [41], frontalin is one of the main pheromones identified in most *Dendroctonus* species [42], such as in *D. frontalis* [43], *D. ponderosae* [44,45], *D. mesoamericanus* [41], and *D. valens* [46]. This last species is the *D. rhizophagus* sibling species, and both coexist in Mexico, performing their life cycle in many places without interfering with each other, since their colonization strategies and host preferences are different. 

Several in vitro assays have documented that the feeding and topical application of JH III in some bark beetle species—such as *Ips confusus*, *Ips pini* [47], *Dendroctonus armandi* [48] and *Dendroctonus jeffreyi* [24,49]—increases the transcription levels from the MVA pathway genes, and in some cases, it also induces the production of ipsdienol or frontalin in the midgut tissue [24,25,26]. Because frontalin and ipsdienol were not previously identified in the gut of *D*. *rhizophagus* [40], we evaluated the potential for the de novo synthesis of frontalin and ipsdienol in pre-emerged adults of this species after phloem feeding and topical exposure to JH III. For this purpose, MVA pathway genes were cloned, molecularly characterized, and their relative expression was measured. In addition, we identified and quantified by Gas Chromatography Mass Spectrometry (GC-MS) the compounds present in the gut of fed males and females of *D. rhizophagus*.

## 2. Results

### 2.1. Identification of MVA Pathway Genes and Phylogenetic Analysis

The highest similarity percentages of all MVA pathway genes from *D. rhizophagus* were found with the sequences from *D. ponderosae* followed by other *Dendroctonus* species. The *MK* gene had the lowest nucleotidic (83%) and aminoacidic (69.8%) similarities, whereas *HMGR* (nt 96%) and AACT (aa 97.6%) had the highest. The similarities obtained with other coleopterans—like *Tribolium castaneum* and *Leptinotarsa decemlineata*—were on average 70% for nucleotides and 69% for amino acids (Table 1). In general, *MK* was the most variable of all MVA pathway genes.

**Figure 1:**
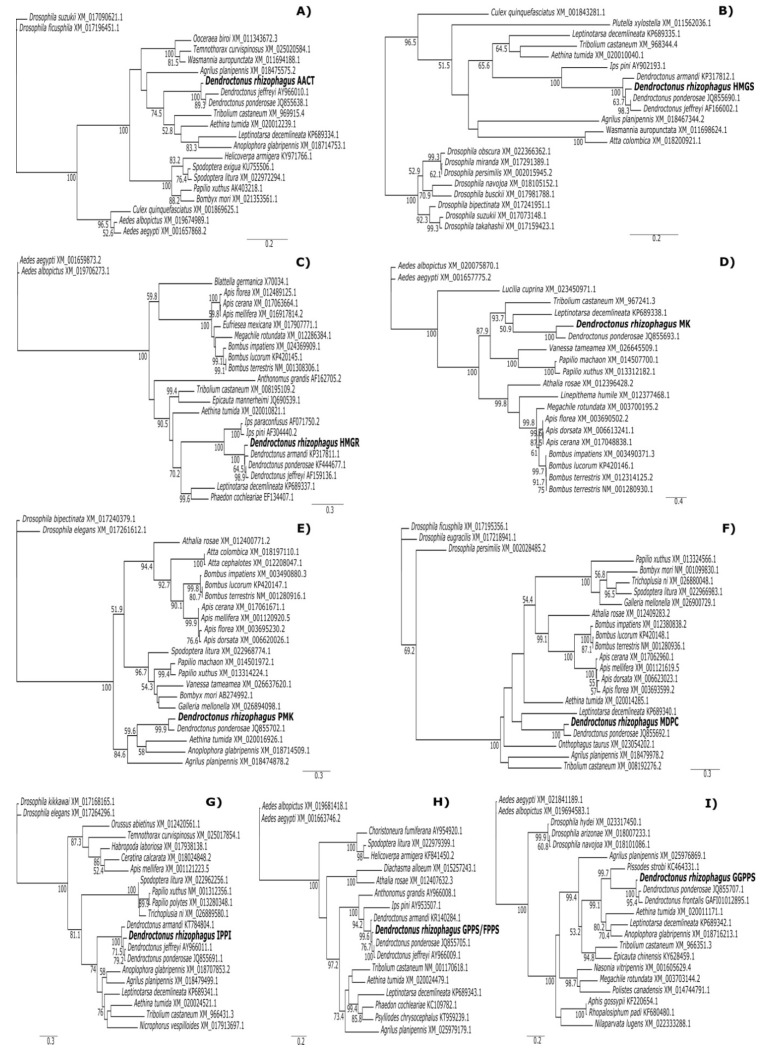
Maximum likelihood trees of MVA pathway genes based on the amino acid sequences of *D. rhizophagus* along with insect sequences obtained from GenBank. Bootstrap values after 1000 replicates are shown at nodes. (**C**) HMGR used the model JTT (+G +I +F) with an estimated proportion of invariable sites (I) of 0.073 and a gamma parameter (G) of 0.575 (−lnL = 14615.13). (**A**) AACT (I = 0.230, G = 0.929, −lnL = 5343.20); (**B**) HMGS (I = 0.242, G = 1.166, −lnL = 6933.67); (**D**) MK (I = 0.075, G = 1.464, −lnL = 9730.43); (**F**) MDPC (I = 0.221, G = 1.139, -lnL = 8370.35); (**H**) GPPS/FPPS (I = 0.068, G = 1.253, −lnL = 8378.21); and (**I**) GGPPS (I = 0.253, G = 1.001, −lnL = 5004.36) used the model LG (+G +I +F); however, (**G**) IPPI (I = 0.150, G = 1.240, −lnL = 5622.99) and (**E**) PMK (I = 0.160, G = 1.459, −lnL = 3924.98) used the model LG (+G +I).

The phylogeny of each of the MVA pathway enzymes of *D. rhizophagus* demonstrated that they integrate consistent groups (Bootstrap values >63%) with the corresponding enzymes of other *Dendroctonus* species. Slight differences between species of this genus are attributable to synonym substitutions that occur mostly in the third position of some triplet codons, but not within or around the motif of each enzyme. Similarly, *Dendroctonus* spp. enzymes are different from those of other bark beetles (e.g., *Ips*) and weevils (e.g., *Pissodes*), but in general, all Coleopterans integrated into a consistent group different from other insect groups in the trees of each protein (Bootstrap values > 63%) (Figure 1).

### 2.2. Molecular Characterization of MVA Pathway Genes

The full-length ORF of putative MVA pathway genes varied from 558 to 2550 bp encoding 185 to 849 amino acids. Among the predictions of physicochemical properties (Table 2), the M.W. ranged from 21.3 to 93.4 kDa, and pI ranged from 6.04 to 8.7. According to the subcellular localization predictions, the MVA pathway’s putative proteins can be in the mitochondria, in the cytoplasm, in the endoplasmic reticulum membrane, in the peroxisome, or extracellular (Table 2). Regarding the secondary structure, five transmembrane helices were determined in the HMGR putative protein. The α-helices and β-sheets numbers predicted for each putative protein were different. The number of α-helices varied from 8 (PMK and IPPI) to 16 (HMGR and GPPS/FPPS), whereas the β-sheets varied from 2 (GPPS/FPPS and GGPPS) to 17 (HMGS and MDPC) (Table 2; Figures S1–S9).

### 2.3. Quantitative Real-Time Polymerase Chain Reaction (RT-qPCR)

**Figure 2:**
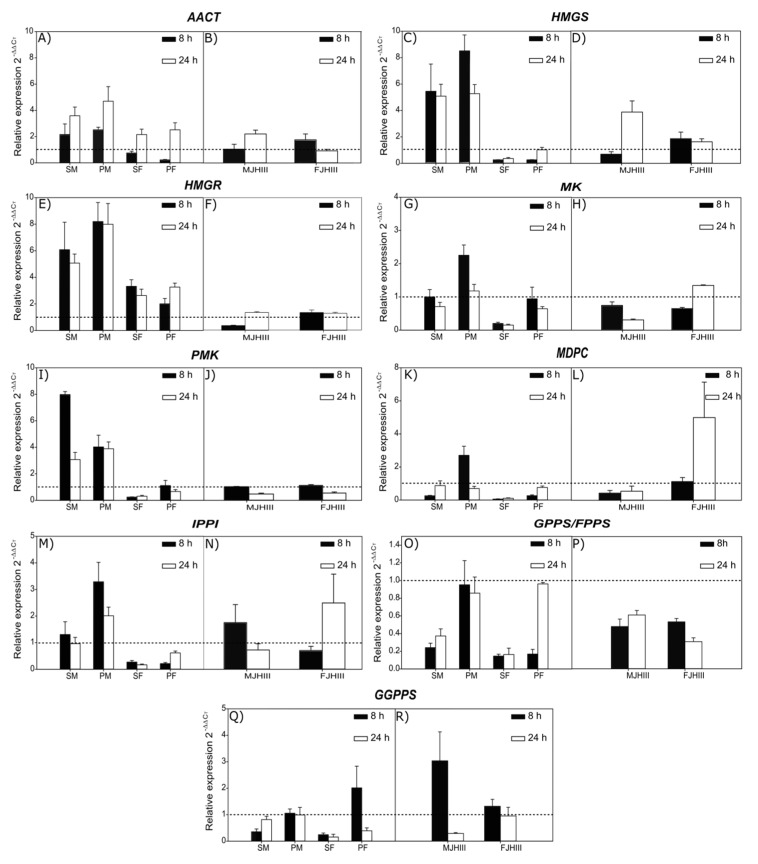
Relative expression of MVA pathway genes calculated by the 2^−^^ΔΔ*C*t^ method in *D. rhizophagus* pre-emerged adults fed on phloem (**A**,**C**,**E**,**G**,**I**,**K**,**M**,**O**,**Q**) or treated with JH III (**B**,**D**,**F**,**H**,**J**,**L**,**N**,**P**,**R**). SM: solitary males, PM: paired males, SF: solitary females, PF: paired females, MJHIII: males treated with JH III, FJHIII: females treated with JH III. Control levels are indicated with the dashed lines; values > 1 on the y-axis (2^−^^ΔΔ*C*t^) indicate upregulation.

Fed males, solitary and in pairs, displayed higher expression levels than females in most genes, with the induction of gene expression being more evident in *AACT*, *HMGS*, *HMGR*, *PMK*, and *IPPI* genes. In the case of insects exposed to JH III, a limited effect in the induction of MVA pathway genes was observed, due to the lack of a clear pattern for any sex or time (Figure 2). Overall, our findings illustrated that the expression levels of fed insects were higher than those from insects stimulated with JH III for almost all MVA pathway genes. 

Regarding the influence of the analyzed factors over expression levels (Tables S1–S4), statistically significant differences were found between sexes in all genes (*p* < 0.01), except for *GGPPS*. Whereas *HMGS* and *HMGR* genes showed significant differences only between sexes (*p* < 0.001), in the rest of the genes, these differences were also found in the other factors: time and condition. The feeding condition (solitary or paired) was not significant in the expression levels of the early genes (*AACT*, *HMGS*, *HMGR*), but it was for the *MK*, *MDPC*, *IPPI*, *GPPS*/*FPPS*, and *GGPPS* genes (*p* < 0.005). Time was only significant for the *AACT*, *MK*, *PMK*, and *GPPS*/*FPPS* genes (*p* < 0.05).

### 2.4. Quantification of Volatile Compounds

**Figure 3:**
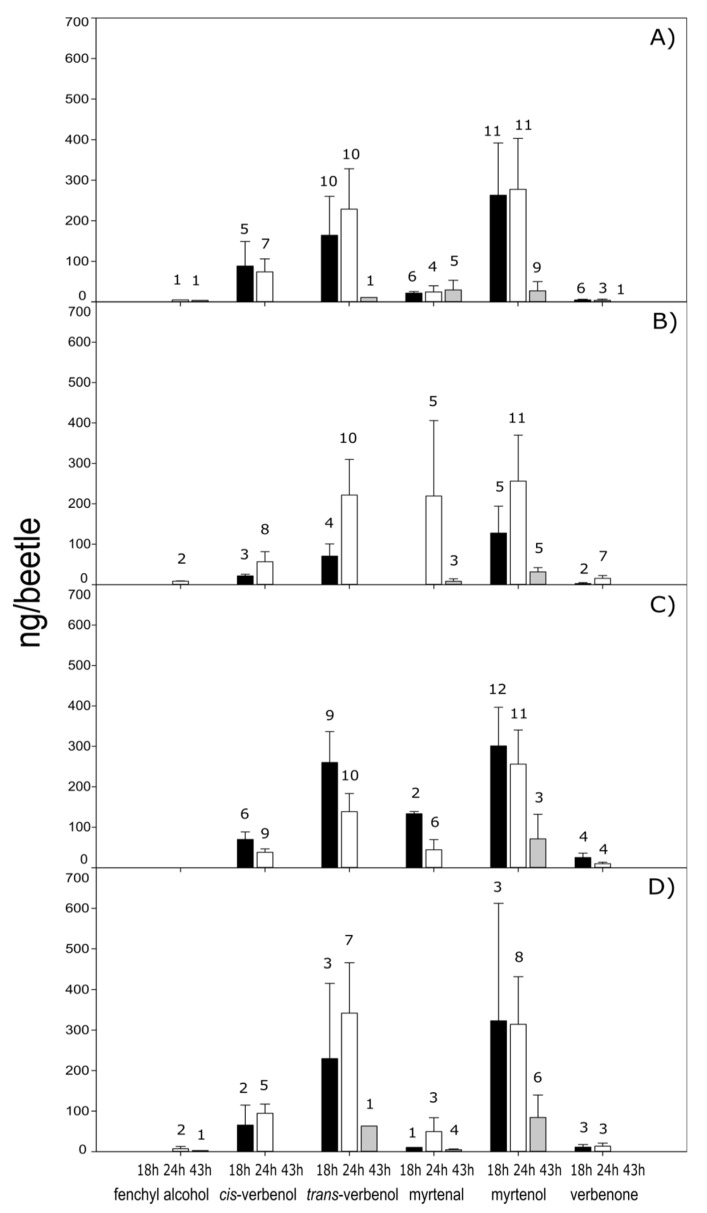
Quantities (mean + SEM) of oxygenated monoterpenes detected in pre-emerged *D*. *rhizophagus* adults fed during 18, 24, and 43 h on the phloem of *Pinus leiophylla*. (**A**) solitary females, (**B**) females paired with males, (**C**) solitary males, (**D**) males paired with females. Numbers above each bar correspond to the number of insects in which the compound was detected out of a maximum of 12 adults tested.

Six oxygenated monoterpenes: fenchyl alcohol, cis-verbenol, trans-verbenol, myrtenal, myrtenol, and verbenone were identified from extracts of dissected guts of phloem-fed *D*. *rhizophagus* females and males, both in solitary or paired conditions, at 18, 24, and 43 h. Higher quantities of oxygenated monoterpenes were recorded either at 18 or 24 h of feeding, whereas few compounds were detected at 43 h (Figure 3). The extracts of dissected guts from the unfed insects showed low quantities of some monoterpenes, while females presented fenchyl alcohol and verbenone. In males, traces of myrtenal, myrtenol, and verbenone were detected (Table 3).

Trans-verbenol and myrtenol were the oxygenated monoterpenes detected in higher quantities in fed insects. Myrtenol was the only compound detected in all treatments, sexes and times. The average quantity of this oxygenated monoterpene at 18 and 24 h varied between 256–323 ng/beetle, except in paired females, where the value reached 127.5 ng/beetle. The trans-verbenol quantities were higher than cis-verbenol ones (≈3 times), with the latter being recorded only at 18 and 24 h in all treatments. Myrtenal was detected at the three feeding times in solitary females and paired males, but the highest quantities were recorded in paired females at 24 h. Finally, fenchyl alcohol and verbenone were detected only at low quantities; the former was recorded at 24 and 43 h both in solitary and paired females, as well as in paired males, whereas verbenone was mainly detected at 18 and 24 h in all treatments (Figure 3).

## 3. Discussion

The phylogenetic analyses of MVA pathway enzymes in *D. rhizophagus* produced well supported topologies (bootstrap values >63%), suggesting the monophyly of these enzymes in *Dendroctonus* species. Phylogenies also showed a general topology congruent with the integrity of taxonomical groups (Figure 1). Despite the low number of sequences of these enzymes available in different databases, the integration of consistent monophyletic groups in each MVA pathway enzyme of *Dendroctonus* spp. suggests that their functions and biochemical characteristics are conserved. In fact, many mutational changes observed in the sequences of genes occurred in the third position of the codons, as suggested by the high frequency of synonym mutations in amino acids located outside the catalytic region. 

The in silico analysis suggested subcellular localizations for all MVA pathway enzymes in *D. rhizophagus*, which partially agrees with those known for other insect or mammal species (Table 2). For example, the mitochondrial prediction of the AACT enzyme in *D. rhizophagus* has also been reported as mitochondrial in human [50], but as cytosolic in *Ostrinia scapulalis* [51]. The HMGS enzyme, inferred as cytoplasmic in *D. rhizophagus*, has been recorded as mitochondrial and cytosolic in humans [52] and rats [53], and as cytosolic in German cockroach, *Blattella germanica* [54] and the Pacific beetle cockroach, *Diploptera punctata* [55]. The HMGR, predicted as an enzyme of the endoplasmic reticulum membrane in *D. rhizophagus*, has also been reported in the same site in mammals [56,57] and insects [58]. The prediction for MK, PMK and MDPC enzymes in *D. rhizophagus* agree with the cytosolic location [59,60] or in peroxisomes [61,62] reported in insects and humans. Lastly, the mitochondrial and cytoplasmic predictions for GPPS/FPPS and GGPPS enzymes in this bark beetle were in concordance with the findings for these enzymes in mammals [63,64] and *Drosophila melanogaster* [65]. The IPPI enzyme of *D. rhizophagus* was the only protein whose predicted location (cytoplasmic) disagrees with those reported in mammals (peroxisomes) [66]. 

The expression analysis showed that phloem feeding and JH III topical application differentially induced several MVA pathway genes in *D. rhizophagus* (Figure 2). Most genes reached higher expression levels in fed rather than stimulated insects, with solitary and paired males showing the highest levels in almost all genes. These findings agree with previous reports on the expression of these genes in bark beetles, which demonstrated that MVA pathway genes presented higher upregulation levels in males than females in the majority of analyzed genes. In addition, male genes are continuously reported as upregulated, whereas female gene expression can be upregulated, downregulated or remain at basal levels (Table 4). With respect to MVA pathway genes in JH III stimulated insects, an irregular expression pattern was displayed, and there was no tendency for a specific sex or time (Figure 2). However, other studies in bark beetles have reported that JH III induced some of these genes, showing high expression levels mainly in males, or at least higher compared to females [25,27,28,48,67,68].

Upregulation was observed mainly in the *AACT*, *HMGS*, *HMGR*, and *PMK* genes in fed males of *D. rhizophagus* in both conditions (solitary and paired) and both times. Females only exhibited upregulation in *AACT* at 24 h. The *HMGR* gene, whose corresponding enzyme synthesizes mevalonate, was the only gene that exhibited upregulation in both fed males and females at both times and conditions (solitary or paired). HMGR is a highly regulated enzyme [69], and its gene is one of the most studied in bark beetles, together with *HMGS*.

These last genes achieved similar expression levels in *D. armandi* males both in phloem-fed insects and JH III stimulated insects [48], but in *I. confusus*, both genes reached higher expression levels in phloem-fed insects than in stimulated insects. In contrast to what was observed in fed insects, neither the enzymatic activity of HMGS, HMGR, and GPPS nor pheromone production was recorded in the JH III stimulated insects of *I*. *confusus*. It has been hypothesized that the deficiency of pheromone production in JH III stimulated insects might be due to the absence of a brain hormone that is only induced by feeding [47]. However, this might also be caused by the presence of a regulatory mechanism mediated by an AMP-activated protein kinase (AMPK) analog to that reported in mammals, in which HMGR enzymatic activity is regulated according to the available energy. A high AMP:ATP ratio induces HMGR phosphorylation by AMPK, which inhibits the enzyme [72]. An analog strategy has been reported in yeasts, where the enzyme is inactivated when the available energy is low [73]. These strategies were presumably developed by convergent evolution and might also be present in insects, whose regulation mechanisms are not yet known [72]. 

The upregulation of *MDPC* and *IPPI* genes observed in paired fed males in both times, as well as in JH III stimulated males at 8 h and females at 24 h, suggests that the synthesis of isopentenyl diphosphate (IPP) and dimethylallyl diphosphate (DMAPP) might occur in the gut of *D*. *rhizophagus*. Nevertheless, whereas both IPP and DMAPP are essential for the de novo production of frontalin or ipsdienol, the expression of *GPPS/FPPS* and *GGPPS* genes recorded in this bark beetle does not reflect the potential for de novo production of these compounds because they do not show a sex-specific expression pattern, nor do they exhibit sustained upregulation over time. Nevertheless, the basal activity of GPPS/FPPS and GGPPS might still allow the synthesis of terpenoid metabolites involved in basic cellular functions, e.g., protein prenylation and glycosylation (dolichols), and the electron transport chain (ubiquinone) [74,75,76].

In those bark beetle species that produce frontalin and ipsdienol, the *GPPS*/*FPPS* and *GGPPS* genes have always been recorded as upregulated in males (Table 4). In *I*. *pini* and *I*. *confusus*, it was demonstrated that the *GPPS* gene is upregulated from 4 to 32 h in fed males [47], whereas it was downregulated in females [32]. The GPPS enzyme and the cytochromes CYP9T2 and CYP9T3 are involved in the final steps of the production of ipsdienol and ipsenol pheromones from myrcene by males of these species [14,77,78]. In *Dendroctonus* species, the final steps of frontalin synthesis have not been described, despite the fact that males or females of several species (e.g., *D. ponderosae*, *D. jeffreyi*, *D. armandi*) produce frontalin either as aggregation, antiaggregation, or sexual pheromone [23,44,49,79]. In particular, it has been reported that the *GGPPS* gene is upregulated from 8 to 72 h in *D. ponderosae* males, which produce frontalin after phloem feeding, allowing us to hypothesize that the enzyme of this gene, together with cytochrome P450 enzymes, are involved in frontalin production through an undetermined mechanism [36,71]. 

Until now, it has been assumed that the upregulation of MVA pathway genes in the gut of bark beetles is directly involved in the synthesis of frontalin or ipsdienol. However, regardless of the observed upregulation of several genes in *D*. *rhizophagus*, our GC-MS analysis did not detect the presence of frontalin or ipsdienol pheromones. This result corresponds with previous findings in the same species from pre-emerged males and females forced to feed on host tissue in the laboratory, as well as in individuals collected from naturally attacked hosts at different stages of colonization [40]. 

It has been suggested that the endogenous production of sexual and aggregation pheromones in bark beetles is not necessarily a fundamental physiological process [18], such is the case of inbreeding species as *Dendroctonus micans* [80], *D*. *punctatus* [81], and presumably *D*. *murrayanae* [82,83], where females are fertilized by their siblings prior to emergence. Field observations of *D*. *rhizophagus* females ovipositing in galleries without a male partner, as well as the presence of sperm in the oviduct of non-emerged females [84], suggest that pheromone production might not be necessary in this species for host colonization and mating. Further studies are needed to expand our understanding of the *D*. *rhizophagus* mating system. However, the lack of frontalin may explain why *D*. *rhizophagus* does not interfere with its sibling species *D*. *valens* in the areas where they coexist in Mexico [85], as they present individual colonization strategies in which *D*. *valens* uses frontalin both as a sexual and an aggregation pheromone [46], as opposed to *D*. *rhizophagus*. The lack of pheromone production by *D. rhizophagus* prevents the colonization of adult trees, which are instead colonized by *D. valens*, confining its attacks to pine regeneration.

However, our GC-MS assays confirmed the abundance of volatile compounds previously identified in *D*. *rhizophagus* gut, such as trans-verbenol and myrtenol. In particular, trans-verbenol was the compound that generated the best electrophysiological response in conspecifics [40].

The production of trans-verbenol and myrtenol is not associated with the MVA pathway activity in insects. These oxygenated monoterpenes are produced from the transformation of the pinenes obtained by the insect through feeding [86,87], or from their accumulation during immature stages and release upon their emergence or colonization establishment [9]. It has been assumed that these compounds are byproducts of a detoxification metabolism of host monoterpenes rather than metabolites synthesized through any metabolic pathway [88]. In fact, recently in *D*. *ponderosae*, it was reported that trans-verbenol is a product of the hydroxylation of (−)-*α*-pinene and (+)-*α*-pinene performed by the action of cytochrome CYP6DE1 [10]. 

## 4. Materials and Methods 

### 4.1. Insect Collection 

On June 2016, pre-emerged unfed adults (fully-melanized brood adults present in dead trees colonized the previous year that are moving outwards from the host tree to emerge) were manually collected from the roots or stem base of *P. leiophylla* seedlings and saplings at Chavarría (N 23°38′01.90′′ W 105°36′00.18′′) and Mil Diez (N 23°48′28.12′′ W 105°24′11.63′′) localities, Pueblo Nuevo municipality, Durango, Mexico. The insects were sexed according to the shape of the seventh abdominal tergite [89] and transported to the laboratory in Magenta™ vessels GA 7 (Magenta Corp, Sigma-Aldrich-Merck, Darmstadt, Germany) under dark conditions at 4 °C.

### 4.2. Treatments Aimed at Induction of MVA Pathway Genes 

To obtain cDNA for the amplification and cloning of MVA pathway genes, solitary females (*n* = 12) and males (*n* = 12), as well as pairs (*n* = 12♀, ♂), were introduced separately into 5.0 mm depth and 6.0 mm diameter holes drilled in fresh logs of uninfested *Pinus leiophylla* to feed for 24 h (feeding treatment). In the case of couples, females were placed first into the holes and males were introduced 4 h later. Both sexes were confined inside the logs for 24 h. The logs were stored at room temperature in dark conditions, and patches of stainless steel mesh were stapled over the drilled holes to prevent insects from escaping. In addition, females (*n* = 12) and males (*n* = 12) were exposed to JH III (Sigma-Aldrich-Merck, Darmstadt, Germany) by applying 6 μg of this hormone dissolved in 0.5 μL of acetone over the ventral area of the abdomen (JH III treatment) with a Hamilton 5 μL syringe (Hamilton Company, Reno, NV, USA) [24,25,28]. Each insect was placed individually in a 1.5 mL microcentrifuge tube and incubated for 24 h at room temperature in the dark.

### 4.3. Total RNA Isolation and cDNA Synthesis 

Six insects of each group from both the fed and JH III treated insects were dissected, in order to obtain the complete alimentary canal. For insects fed in pairs (♀, ♂), each sex was processed individually. The tissues of each group were pooled and macerated in TRI Reagent^®^ Solution (Ambion™, Thermo Fisher Scientific, Waltham, MA, USA), frozen in liquid nitrogen, and kept at −80 °C until being processed with the RiboPure™ RNA Purification Kit (Ambion™ Thermo Fisher Scientific) according to the manufacturer’s protocol. RNA concentration and purity (A_260_/A_280_ ratio) were measured with a NanoDrop™2000 spectrophotometer (Thermo Fisher Scientific). RNA integrity and non-DNA contamination were verified on 1% denatured gels of agarose UltraPure (Invitrogen, Carlsbad, CA, USA) and visualized in a UV transilluminator (Alpha Innotech Corporation, San Leandro, CA, USA). Two μg of RNA were used for cDNA synthesis with High-Capacity RNA-to-cDNA™ Kit (Applied Biosystems™ by Thermo Fisher Scientific) according to the manufacturer’s protocol. The synthesized cDNA was stored at −20 °C until further use [90,91].

### 4.4. Amplification and Cloning of MVA Pathway cDNAs

In order to obtain the full-length cDNA sequences, PCR amplifications were performed in a thermocycler (Techne, Cambridge, UK) in a total reaction volume of 20 μL: 0.2 mM dNTPs, 2 mM MgCl_2_, 0.5 μM of each primer, 0.5 μM BSA (*AACT*, *MK*, *MDPC*, *GGPPS*), DMSO 5% (with BSA for *HMGS*, *HMGR*, *GPPS*/*FPPS*), 2 μL cDNA and 1.25 U Thermo Scientific™ DreamTaq™ DNA Polymerase (Thermo Fisher Scientific). An initial denaturation step at 94 °C for 3 min was followed by 35 cycles of denaturation at 94 °C for 40 s, annealing for 50 s, and extension at 72 °C, with a final extension of 30 min for all transcripts. The annealing temperature and the extension time of each cDNA are reported in the supporting information (Table S5). 

Primers (Table S5) were designed according to the sequences obtained from the *D. rhizophagus* transcriptome [92]. The amplicons were purified with GFX PCR DNA and the Gel Band Purification Kit (Illustra GE Healthcare, Chicago, IL, USA), ligated to pGEM^®^-T Easy Vector System I (Promega, Wisconsin, USA), and cloned into chemically competent *Escherichia coli* DH5α cells. Plasmid DNA was extracted by the alkaline lysis method [93] and sequenced in a 3730xl DNA Analyzer (Applied Biosystems, CA, USA) at Macrogen Inc., Seoul, Korea.

### 4.5. In Silico Characterization of MVA Pathway Genes

Blast searches for full-length sequences of each gene and putative proteins were made against the NCBI GenBank [94] and UniProt databases for their identification and comparison (%similarity) with previously reported coleopteran sequences. Nucleotide sequences were translated with the ExPASy Translate tool from the SIB (Swiss Institute of Bioinformatics) [95], and the amino acid sequences were used to predict physicochemical properties, including molecular weight (M.W.) and isoelectric point (pI) using the ProtParam program [96]. Probable subcellular localizations were estimated with the TargetP program (non-plant proteins) [97] and ProtComp v. 9.0 (eukaryotic proteins). Moreover, amino acid sequences were checked for transmembrane helices with TMHMM 2.0 [98]. The ESPript program was used to assign a possible secondary structure to putative *D. rhizophagus* MVA pathway proteins based on crystal structure data from the Protein Data Bank (PDB) [99].

### 4.6. Phylogenetic Analysis

The complete sequences of the MVA pathway putative enzymes were aligned using Clustal v. 1.2.1 [100] with sequences deposited in the GenBank from other insect groups. ProtTEST v2.4 [101] was used to select the best-fitting protein evolution model for each gene based on the Akaike information criterion. The LG +G +I +F model was selected for almost all putative proteins, with the exception of HMGR (JTT +G +I +F). Maximum likelihood (ML) phylogenetic analyses were performed in PhyML [102] (http://atgc.lirmm.fr/phyml/) with the determined model for each enzyme. Node support was assessed by 1000 bootstrap replicates. Dipteran sequences were used as the outgroup (accession numbers are given in the corresponding trees).

### 4.7. RT-qPCR Assays

Quantitative real-time PCR (RT-qPCR) was used to obtain the relative expression of MVA pathway genes in the *D. rhizophagus* gut. All experimental procedures related to qPCR were performed according to the Minimum Information for Publication of Quantitative Real-Time PCR Experiments (MIQE) guidelines (Table S6) [103]. For this, solitary females (*n* = 18), solitary males (*n* = 18), as well as pairs (*n* = 18) formed by one male and one female, were fed separately in the dark both for 8 and 24 h. In the case of pairs, each sex was processed individually. Unfed solitary insects (males and females) were included as controls. On the other hand, males and females stimulated with JH III were set independently into 1.5 microcentrifuge tubes in the dark for 8 and 24 h. Insects of both sexes who were treated with 0.5 μL of acetone during both exposure times were included as control groups. As these times elapsed, all insects were killed and dissected for total RNA isolation and cDNA synthesis, as described above. Three biological replicates were conduced, each one with six guts, for each treatment in the qPCR assays. 

Real-time PCR reactions were carried out with a Step One™ Real-Time PCR System (Applied Biosystems). Primers and a TaqMan^®^ hydrolysis probe for each gene were designed by Applied Biosystems (Table S5); probes were labeled at the 5’ end with the reporter dye 6-carboxyfluorescein (FAM) and a non-fluorescent quencher molecule group MGB (Minor Groove Binder) at the 3′ end. Each reaction contained 900 nM of each primer, 250 nM of the TaqMan^®^ probe, TaqMan^®^ Universal Master Mix II and 5 μL cDNA in a final volume of 20 μl. Three technical replicates were performed for each biological replicate. The following standard manufacturer’s amplification conditions were used: 50 °C for 2 min, 95 °C for 10 min, and 40 cycles at 95 °C for 15 s and 60 °C for 60 s. The efficiency and validation (R^2^) of the qPCR were assessed for each gene with a linear regression analysis of the mean values of the quantification cycles (Cq) from different dilutions of cDNA (efficiency = ((10^−1/slope^ − 1) × 100)). The mean slope value was −3.22 ± 0.32 (s.d.) and the R^2^ values were >0.96. All amplicons were visualized in 1% agarose gel electrophoresis to evaluate the specificity. Relative expression values were calculated according to the 2^−ΔΔ*C*t^ method [104], using *CYP4G55v1* as the reference gene for normalizing expression levels [90,91,105,106,107].

### 4.8. Statistical Analysis

A three-way ANOVA was used for the analysis of the following factors: sex (male-female), condition (solitary-paired), and time (8 and 24 h) in fed insects; and a two-way ANOVA for the analysis of the following factors: sex (male-female) and time (8 and 24 h) in insects treated with JH III. Tukey’s test was performed for pairwise comparisons between factors. The 2^−ΔΔ*C*t^ values were used for relative expression plots and statistical analysis with SigmaPlot 12.0 and SigmaStat v. 3.5 software (Systat Software Inc., San Jose, CA, USA).

### 4.9. Quantification of Volatile Compounds

Terpenoid compounds present in the gut of *D*. *rhizophagus* males and females were sampled and quantified in: (1) unfed pre-emerged insect; (2) solitary fed males and (3) solitary fed females; (4) fed males paired with females; and (5) fed females paired with males. The unfed pre-emerged insects were immediately processed for analysis by gas chromatography—mass spectrometry (GC-MS). The insects used in the other treatments (2–5) were confined during 18 and 24 h into uninfested *Pinus leiophylla* fresh logs. These times were selected because a preliminary analysis demonstrated that in this period, in laboratory conditions, the production of volatiles by insects is better than in shorter times (<15 h). In addition, to follow up the production of volatiles, these were also recorded and quantified at 43 h. For insects in pairs, females were first introduced alone into the bark during 4 h, and afterwards, a male was introduced with each female and both stayed inside for another 18, 24, and 43 h. Twelve replicates were collected for each treatment and feeding time. All insects were set individually into 0.25 mL glass inserts (SUPELCO, Sigma-Aldrich Corp., Milwaukee, WI, USA) containing ~0.3 mg of the adsorbent Porapak Q, during 24 h in dark conditions at room temperature in a stream of purified air, in order to collect the volatiles released by them [40,108]. Later, each insect was removed from its vial and dissected to obtain the gut. Volatiles were extracted from the Porapak Q and from the gut tissue with 100 μL of hexane spiked with 3.8 ng/μL of cycloheptanone (99 purity, Sigma-Aldrich Corp.) as an internal quantitative standard. The tissue was gently macerated against the glass insert wall and reserved for 15 min for passive extraction at room temperature. Later, the total extract was transferred into a clean glass insert and kept at −20 °C. Negative control extracts were also collected from glass inserts in which no beetles were introduced. 

One microliter of each extract was analyzed by GC-MS (7820A GC System-5975 Series MSD Agilent Technologies, Santa Clara, CA, USA) in splitless mode with a (5%-Phenyl)-methylpolysiloxane column (Agilent J&W HP-5ms 30 m × 0.25 mm × 0.25 μm). The temperature program of the GC oven was 50 °C for 1 min, 2 °C/min to 100 °C, then 16 °C/min to 200 °C. The quantification of each compound was made by internal standardization method and converted to nanograms based on the relative responses obtained from serial dilutions of synthetic standards [fenchyl alcohol (96% purity, Sigma-Aldrich Corp., Milwaukee, WI, USA), myrtenal (98% purity, Sigma-Aldrich Corp.), myrtenol (95% purity, Sigma-Aldrich Corp.), cis-verbenol (95% purity, Sigma-Aldrich Corp.), trans-verbenol (95% purity, PheroTech, Delta, BC, Canada), and verbenone (99% purity, Sigma-Aldrich Corp.). The identification of each compound from the extracts was accomplished by matching the retention times of synthetic standards and mass spectra searches with the NIST Mass Spectral Search Program (version 2.2) for the NIST/NIH/EPA Mass Spectral Library.

## 5. Conclusions

In brief, this study is an effort to determine whether *D. rhizophagus* engages in the de novo synthesis of pheromones such as frontalin and ipsdienol through the MVA pathway. According to the expression patterns observed in *GPPS*/*FPPS* and *GGPPS* genes, it is unlikely that frontalin or ipsdienol are produced in this species, despite *D*. *rhizophagus* males presenting higher expression levels than females, as has been reported in other bark beetle species that produce these pheromones [27,32,48,67]. These findings were also supported by the GC-MS analysis, which did not record the present of these compounds. The information obtained in this study is relevant because it allow us to understand how two species have divided their ecological niches to co-exist in Mexican Forests, as their chemical ecology diverges. Additionally, further studies are needed to reveal the MVA pathway regulatory mechanisms that govern the production of terpenoid pheromones in different bark beetle species.

## Figures and Tables

**Table 1:** Nucleotidic and aminoacidic similarities from mevalonate (MVA) pathway sequences isolated from *D*. *rhizophagus* compared with previously reported *Dendroctonus* species and other coleopteran sequences. (NCBI, National Center of Biotechnology Information).

Gene	Species	Blast NCBI GenBank No.	% Similarity	Blast UniProt UniProt No.	% Similarity
*AACT*	*Dendroctonus ponderosae*	JQ855638.1	93	I1VJ17	97.6
	*Dendroctonus jeffreyi*	AY966010.1	92	Q56CY6	91.5
	*Leptinotarsa decemlineata*	KP689334.1	72	A0A0H4IRH6	81.1
	*Tribolium castaneum*	XM_969915.4	72	D6WAN7	79.9
*HMGS*	*Dendroctonus ponderosae*	JQ855690.1	95	I1VJ69	96.7
	*Dendroctonus jeffreyi*	AF166002.1	94	Q9NDA8	93.7
	*Dendroctonus armandi*	KP317812.1	92	A0A0F7LEZ1	94.5
	*Ips pini*	AY902193.1	72	Q5EE42	75.9
	*Leptinotarsa decemlineata*	KP689335.1	71	A0A0H4J592	67.6
	*Tribolium castaneum*	XM_968344.4	71	D6WTE1	65.9
*HMGR*	*Dendroctonus ponderosae*	KF444677.1	96	U5ZZ40	96.8
	*Dendroctonus jeffreyi*	AF159136.1	95	Q9N6G8	96.3
	*Dendroctonus armandi*	KP317811.1	93	A0A0F7LG51	95.4
	*Ips paraconfusus*	AF071750.2	75	Q9XY99	78.5
	*Ips pini*	AF304440.1	74	Q95WT1	78.1
	*Leptinotarsa decemlineata*	KP689337.1	72	A0A0H4ISG3	66.6
	*Tribolium castaneum*	XM_008195109.2	69	A0A139WIA9	68.3
*MK*	*Dendroctonus ponderosae*	JQ855693.1	83	I1VJ72	69.8
	*Leptinotarsa decemlineata*	KP689338.1	*-*	A0A0H4IUW0	41.8
*PMK*	*Dendroctonus ponderosae*	JQ855702.1	91	I1VJ81	89.1
	*Leptinotarsa decemlineata*	KP689339.1	68	A0A0H4IRI1	59.2
*MDPC*	*Dendroctonus ponderosae*	JQ855692.1	93	I1VJ71	92
	*Leptinotarsa decemlineata*	KP689340.1	68	A0A0H4J596	61.6
	*Tribolium castaneum*	XM_008192276.2	66	D6WE42	64.4
*IPPI*	*Dendroctonus ponderosae*	JQ855691.1	94	I1VJ70	92.9
	*Dendroctonus jeffreyi*	AY966011.1	94	Q56CY5	92.9
	*Dendroctonus armandi*	KT784804.1	86	A0A0N9H5S7	89.6
	*Leptinotarsa decemlineata*	KP689341.1	71	A0A0H4IVW7	70.1
	*Tribolium castanem*	XM_966431.3	65	D6WP58	76.6
*GPPS/FPPS*	*Dendroctonus ponderosae*	JQ855705.1	95	I1VJ84	96.3
	*Dendroctonus jeffreyi*	AY966009.1	94	Q56CY7	94.9
	*Dendroctonus armandi*	KR140284.1	93	A0A0N9EI79	96
	*Ips pini*	AY953507.1	72	Q58GE9	72.1
	*Tribolium castanem*	NM_001170618.1	70	D6WSE5	70.4
	*Leptinotarsa decemlineata*	KP689343.1	66	A0A0H4IUW5	55.5
*GGPPS*	*Dendroctonus ponderosae*	JQ855707.1	94	I1VJ86	95.3
	*Dendroctonus frontalis*	GAFI01012895.1	93	T1DSZ4	94.6
	*Leptinotarsa decemlineata*	KP689342.1	72	A0A0H4ISG7	75.7
	*Tribolium castaneum*	XM_966351.3	71	D6WD18	68.3

--- No significant similarity found.

**Table 2:** Physicochemical properties, cellular localization, and secondary structure elements predicted for *D*. *rhizophagus* MVA pathway’s putative proteins.

Protein	ORF ^1^ (bp) Accession No.	Aa ^2^	M.W. ^3^ (kDa)	pI ^3^	Subcellular Localization Predictions		Secondary Structure Elements		
					TargetP	ProtComp 9.0	TMH ^5^	α-H ^6^	β-S ^6^
AACT	1239 MK387135	412	43.2	8.64	0.458 mTP ^4^, 0.036 SP ^4^, 0.441 Other	Mitochondrial	No TMH	11	16
HMGS	1374 MK387139	457	50.7	6.04	0.017 mTP, 0.643 SP, 0.648 Other	Cytoplasmic	No TMH	15	17
HMGR	2550 MK387137	849	93.4	8.07	0.124 mTP, 0.471 SP, 0.398 Other	Membrane bound Endoplasmic Reticulum	5 TMH	16	11
MK	1308 MK387142	435	48.3	6.17	0.497 mTP, 0.046 SP, 0.579 Other	Cytoplasmic	No TMH	11	13
PMK	558 MK387143	185	21.3	6.89	0.092 mTP, 0.077 SP, 0.881 Other	Peroxisomal	No TMH	8	5
MDPC	1161 MK387140	386	42.9	7.01	0.058 mTP, 0.275 SP, 0.748 Other	Extracellular	No TMH	11	17
IPPI	759 MK387141	252	28.9	6.31	0.348 mTP, 0.133 SP, 0.404 Other	Cytoplasmic	No TMH	8	9
GPPS/FPPS	1290 MK387138	429	49.5	8.7	0.778 mTP, 0.063 SP, 0.223 Other	Cytoplasmic/Mitochondrial	No TMH	16	2
GGPPS	894 MK387136	297	34.3	6.33	0.108 mTP, 0.091 SP, 0.855 Other	Cytoplasmic	No TMH	15	2

^1^ Open Reading Frame. ^2^ Number of amino acids defined by the ExPASy Translate tool. ^3^ Molecular Weight and Isoelectric point predicted with ProtParam. ^4^ mTP: mitochondrial, SP: secretory pathway. ^5^ Predicted number of transmembrane helices (TMH) by the TMHMM Server v. 2.0. ^6^ Number of α-helices (α-H) and β-sheets (β-S) assigned by the ESPript 3.0 program.

**Table 3:** The range of oxygenated monoterpenes quantities (ng/beetle) extracted from the gut of pre-emerged unfed *D*. *rhizophagus*.

Compound	Female	Male
fenchyl alcohol	0.01–0.04 ^1^ 2/12 ^2^	- 0/12
cis-verbenol	- 0/12	- 0/12
trans-verbenol	- 0/12	- 0/12
myrtenal	- 0/12	0.007 1/12
myrtenol	- 0/12	0.03–0.1 3/12
verbenone	0.002–0.003 2/12	0.002–0.05 3/12

^1^ Quantity range (ng/beetle). ^2^ Ratio between number of insects in which the compound was detected and the total number of insects tested.

**Table 4:** Previous reports of relative expression analysis of bark beetle MVA pathway genes after feeding on phloem or JH III treatments.

Gene	Species	Male	Female	Technique	Treatment	Reference
*AACT*	*I. pini*	U	U*	RT-qPCR	Feeding	[32]
	*I. pini*	C	U*	RT-qPCR	JH III	[70]
*HMGS*	*I. pini*	U	U*	RT-qPCR	Feeding	[32]
	*I. pini*	U	U	RT-qPCR	JH III	[70]
	*I. pini*	U*	U	RT-qPCR	JH III	[67]
	*D. jeffreyi*	U*	C	Northern blot	JH III	[27]
	*D. armandi*	U*	U	RT-qPCR	Feeding	[48]
	*D. armandi*	U*	C	RT-qPCR	JH III	[48]
	*I. confusus*	U	NR	RT-qPCR	Feeding	[47]
	*I. confusus*	U	NR	RT-qPCR	JH III	[47]
*HMGR*	*I. paraconfusus*	U*	U	Northern blot	JH III	[68]
	*I. pini*	U*	U	Northern blot	JH III	[25]
	*I. pini*	U	U*	RT-qPCR	Feeding	[32]
	*I* *. pini*	U	U*	RT-qPCR	JH III	[70]
	*I. confusus*	U	NR	RT-qPCR	Feeding	[47]
	*I. confusus*	U	NR	RT-qPCR	JH III	[47]
	*D. jeffreyi*	U*	C	Northern blot	JH III	[28]
	*D. ponderosae*	U*	U	RT-qPCR	Feeding	[71]
	*D. armandi*	U*	U	RT-qPCR	Feeding	[48]
	*D. armandi*	U*	C	RT-qPCR	JH III	[48]
*MK*	*I. pini*	U*	C	RT-qPCR	JH III	[70]
*MDPC*	*I. pini*	U*	U	RT-qPCR	Feeding	[32]
	*I. pini*	U	U*	RT-qPCR	JH III	[70]
*IPPI*	*I. pini*	U	U*	RT-qPCR	Feeding	[32]
	*I. pini*	U	U*	RT-qPCR	JH III	[70]
*GPPS/FPPS*	*GPPS I. pini*	U*	D	RT-qPCR	Feeding	[32]
	*GPPS I. pini*	U	U*	RT-qPCR	JH III	[70]
	*FPPS I. pini*	U*	D	RT-qPCR	Feeding	[32]
	*FPPS I. pini*	U*	**D**	RT-qPCR	JH III	[70]
	*D. ponderosae*	U*	C	RT-qPCR	Feeding	[36]
	*GPPS I. confusus*	U	NR	RT-qPCR	Feeding	[47]
	*GPPS I. confusus*	U	NR	RT-qPCR	JH III	[47]
*GGPPS*	*D. ponderosae*	U*	C	RT-qPCR	Feeding	[71]

U: Upregulated (U*: Sex with the highest expression levels) D: Downregulated C: Control level NR: Not reported.

## Supplementary Materials

Supplementary materials can be found at https://www.mdpi.com/1422-0067/20/16/4011/s1. References [109,110,111,112,113,114,115,116,117,118,119,120,121,122] are cited in the supplementary figures.

## Abbreviations

MVA	Mevalonate
RT-qPCR	Quantitative real-time Polymerase Chain Reaction
GC-MS	Gas Chromatography Mass Spectrometry
