# Supplementary material for: The Differential Expression of Mevalonate Pathway Genes in the Gut of the Bark Beetle Dendroctonus rhizophagus (Curculionidae: Scolytinae) Is Unrelated to the de Novo Synthesis of Terpenoid Pheromones
Source: Int J Mol Sci. 2019 Aug 17;20(16):4011. doi: 10.3390/ijms20164011 (PMC6721070; doi:10.3390/ijms20164011)
Supplement: Supplementary file 1 [file ijms-20-04011-s001.zip › Supplementary Figures IJMS.pdf]

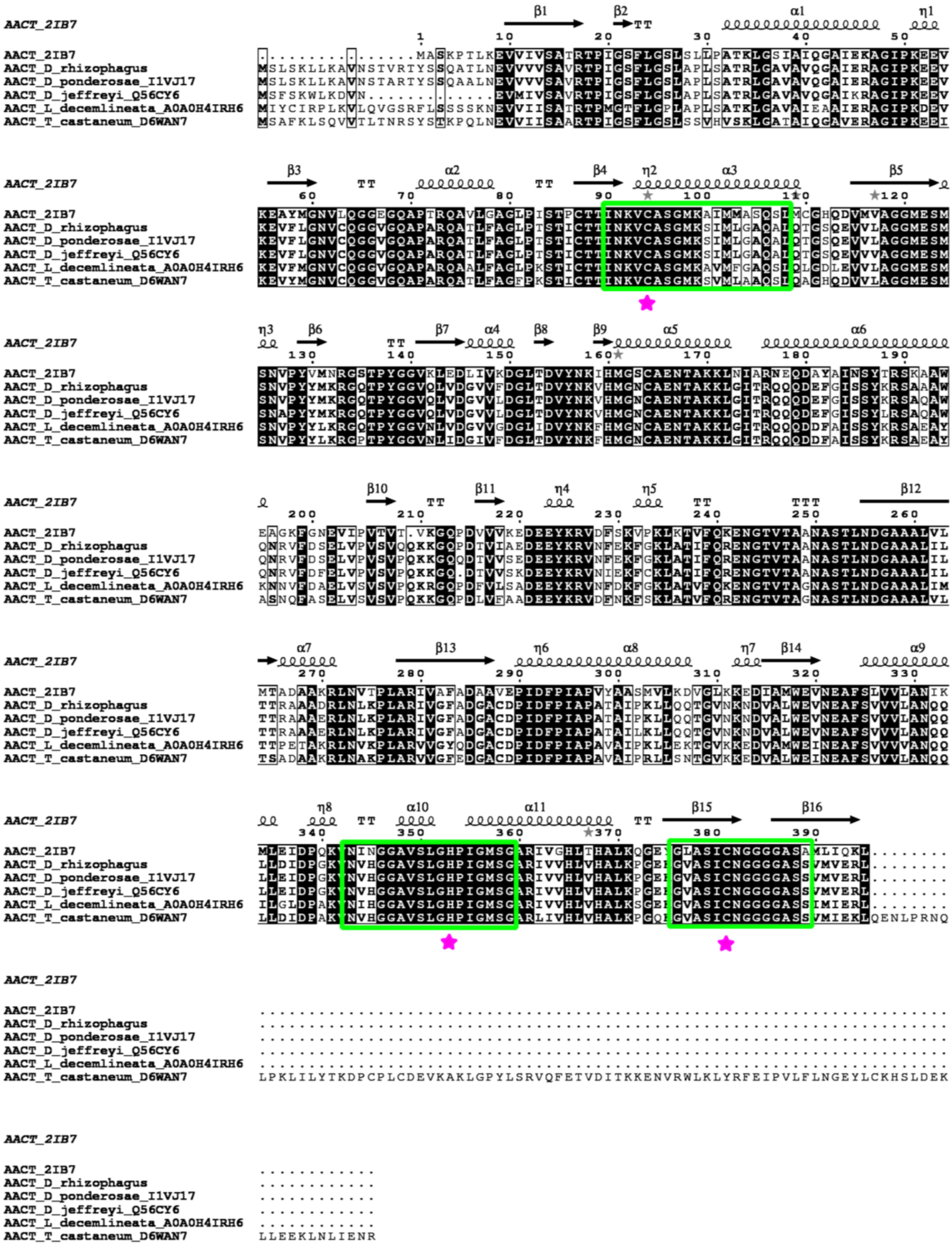

Figure S1. Multiple sequence alignment and assignment of secondary structure elements to acetoacetyl-CoA thiolase (AACT) by ESPript 3.0, according to the *Homo sapiens* protein structure (PDB: 2IB7). The multiple alignments included genus *Dendroctonus* and other coleopteran sequences. Conserved motifs (green boxes) and amino acids involved in the catalytic activity (★) were manually tagged according to previous studies [51] and ExPASy-PROSITE server (http://prosite.expasy.org/).

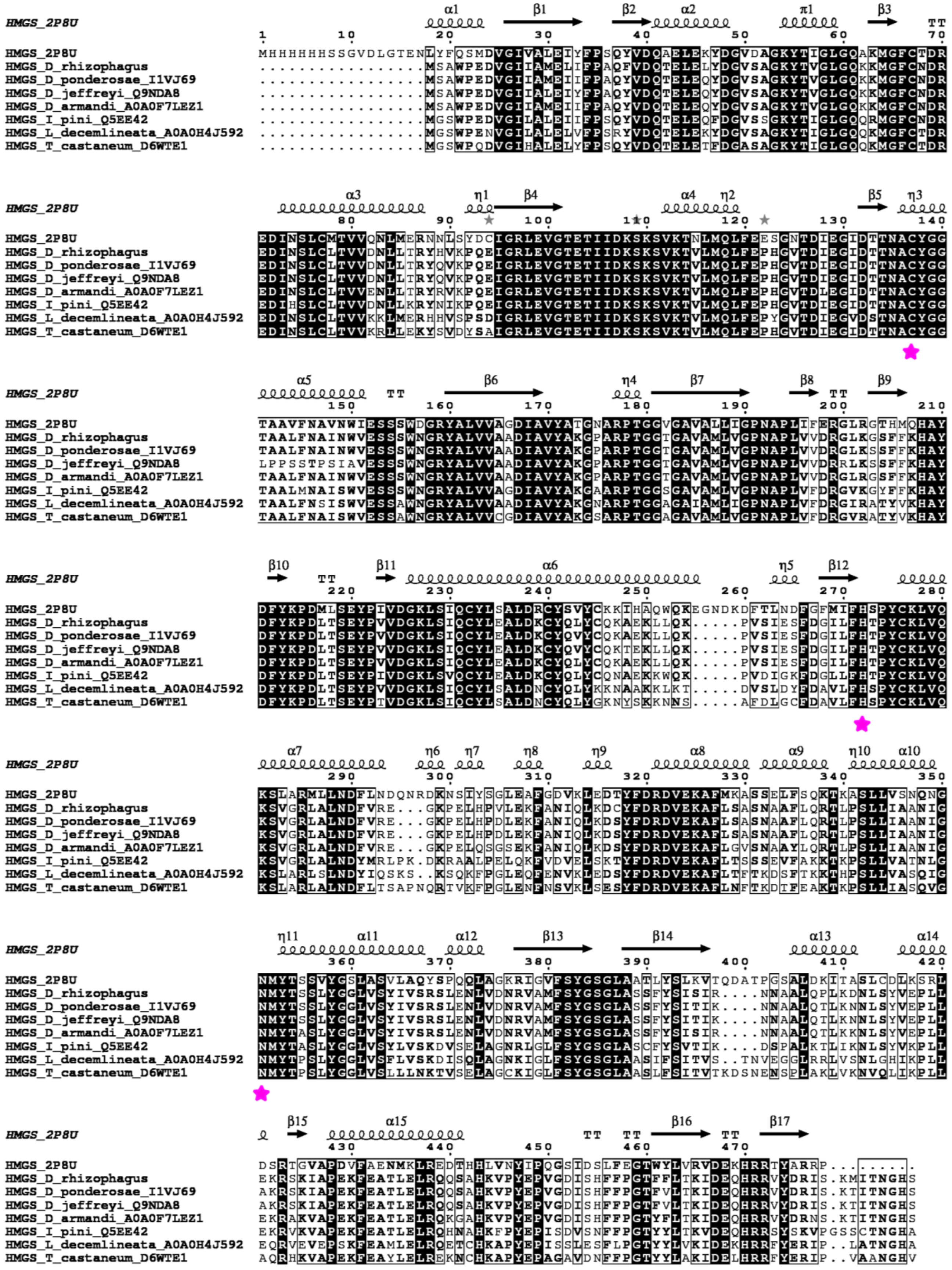

Figure S2. Multiple sequence alignment and assignment of secondary structure elements to 3-hydroxy-3-methylglutaryl-CoA synthase (HMGS) by ESPript 3.0, according to the *Homo sapiens* protein structure (PDB: 2P8U). The multiple alignments included genus *Dendroctonus* and other coleopteran sequences. Conserved amino acids involved in the catalytic activity (⋆) were manually tagged according to previous studies [109] and ExPASy-PROSITE server (http://prosite.expasy.org/).

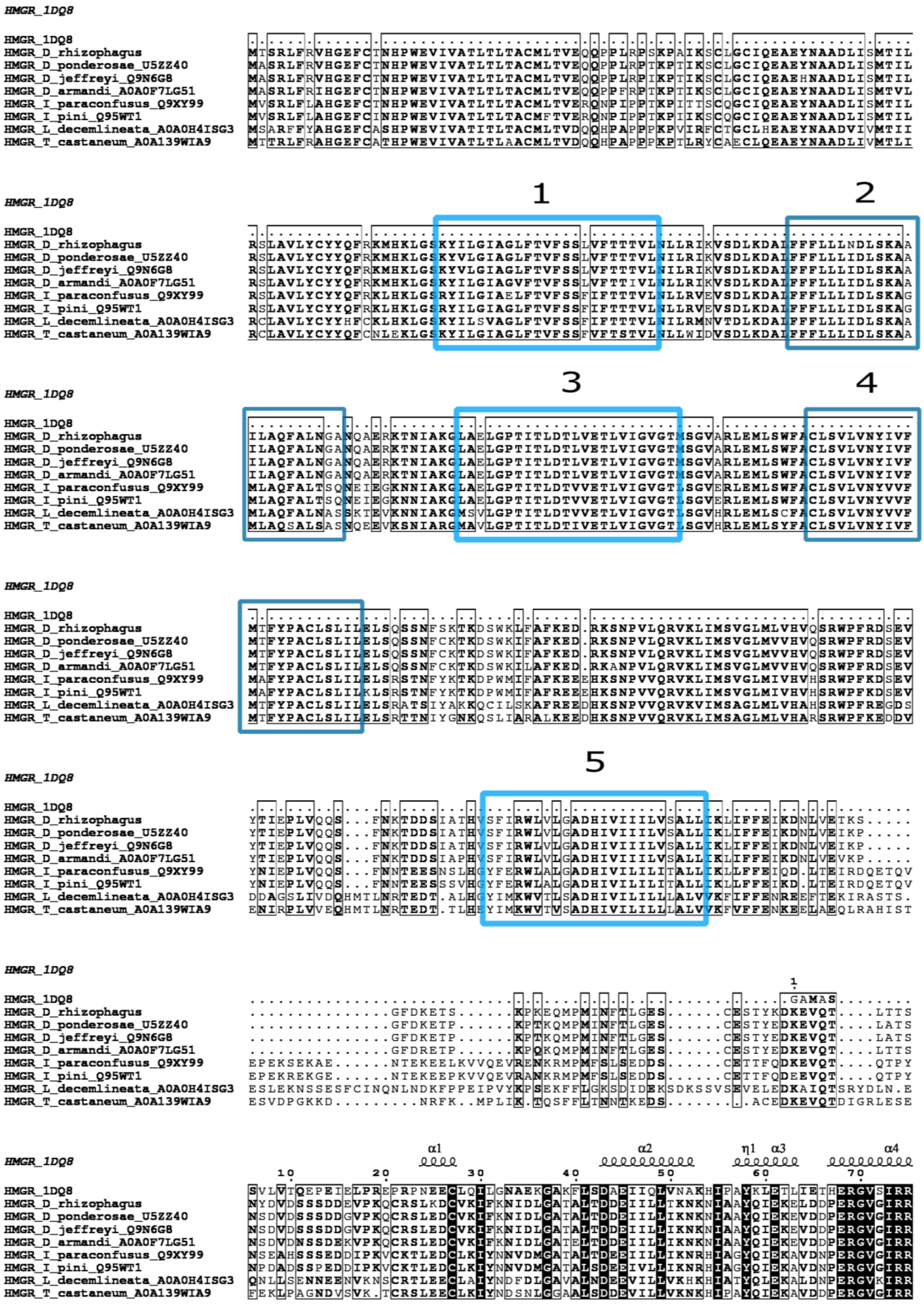

HMGR_1DQ8
HMGR_D_rhizophagus
HMGR_D_ponderosae_U5ZZ40
HMGR_D_jeffreyi_Q9N6G8
HMGR_D_armandi_A0A0F7LG51
HMGR_I_paraconfusus_Q9XY99
HMGR_I_pini_Q95WT1
HMGR_L_decemlineata_A0A0H4ISG3
HMGR_T_castaneum_A0A139WIA9
1
2
3
4
5
α1
α2
η1
α3
α4
10
20
30
40
50
60
70

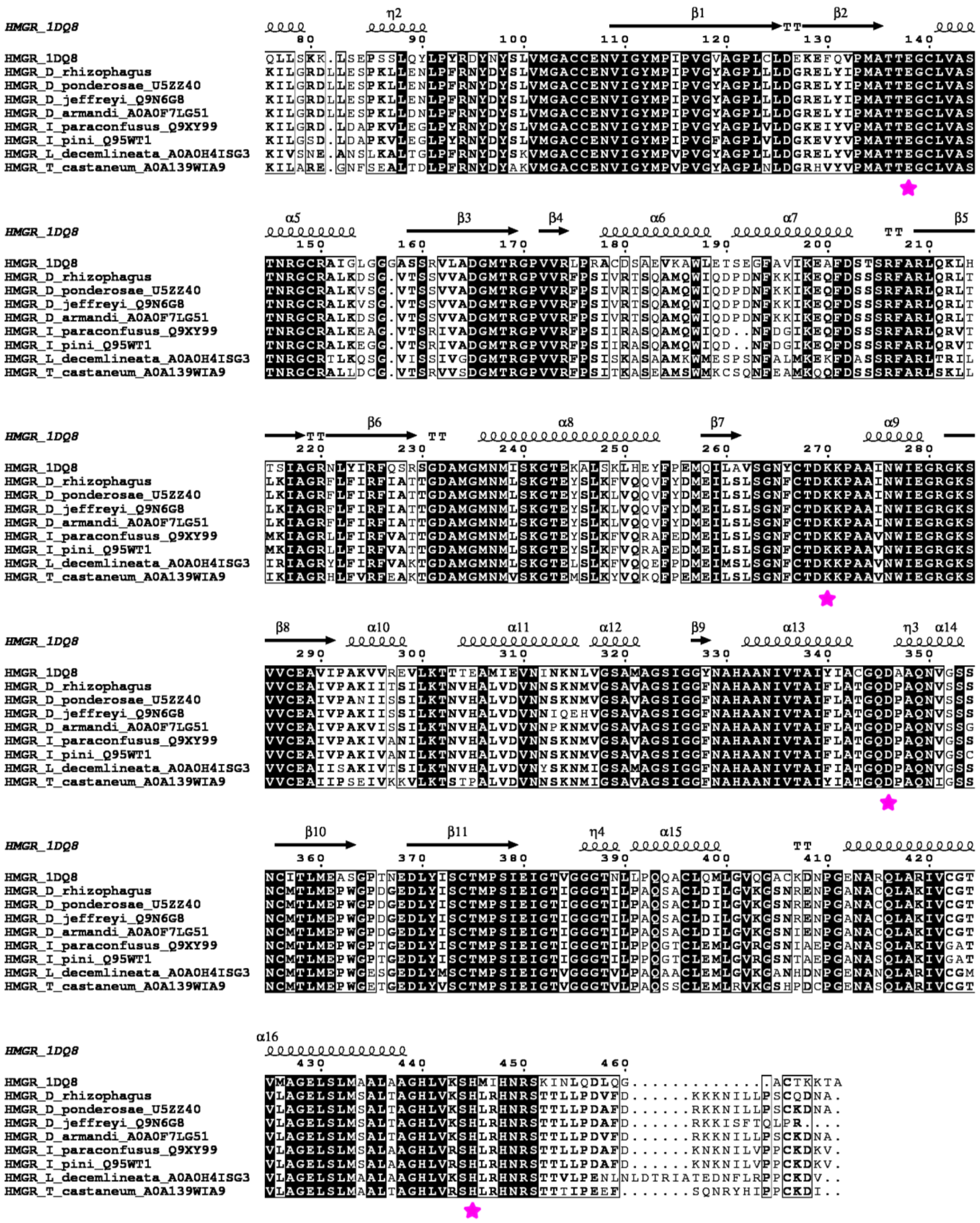

Figure S3. Multiple sequence alignment and assignment of secondary structure elements to 3-hydroxy-3-methylglutaryl-CoA reductase (HMGR) by ESPript 3.0, according to the *Homo sapiens* protein structure (PDB: 1DQ8). The multiple alignments included genus *Dendroctonus* and other coleopteran sequences. TMHMM Server v. 2.0 predicted five transmembrane helices (blue boxes). Conserved amino acids involved in the catalytic activity (★) were manually tagged according to previous studies [110] and ExPASy-PROSITE server (http://prosite.expasy.org/).

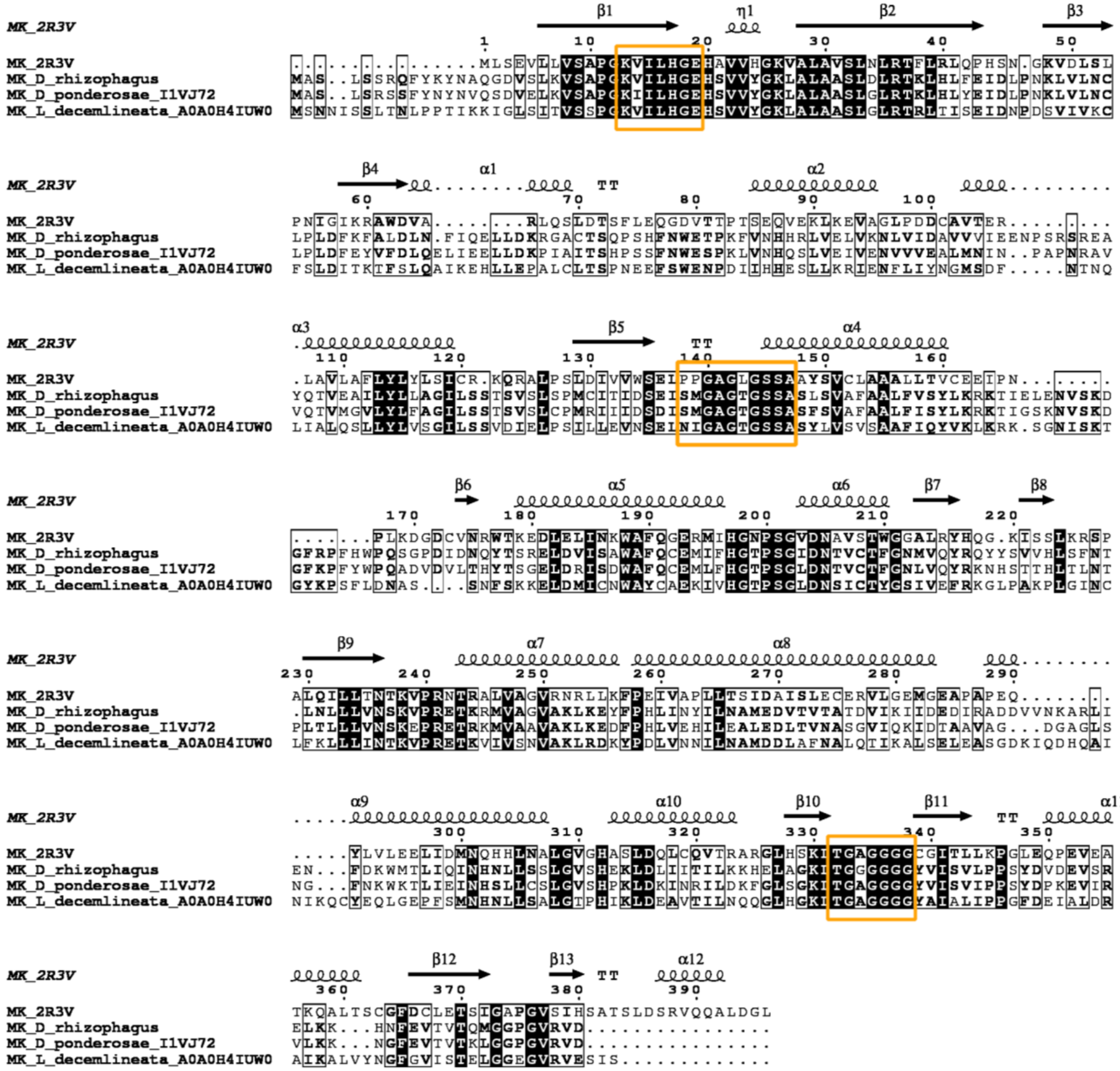

Figure S4. Multiple sequence alignment and assignment of secondary structure elements to mevalonate kinase (MK) by ESPript 3.0, according to the *Homo sapiens* protein structure (PDB: 2R3V). The multiple alignments included genus *Dendroctonus* and other coleopteran sequences. Conserved motifs (orange boxes) were manually tagged according to previous studies [111–113].

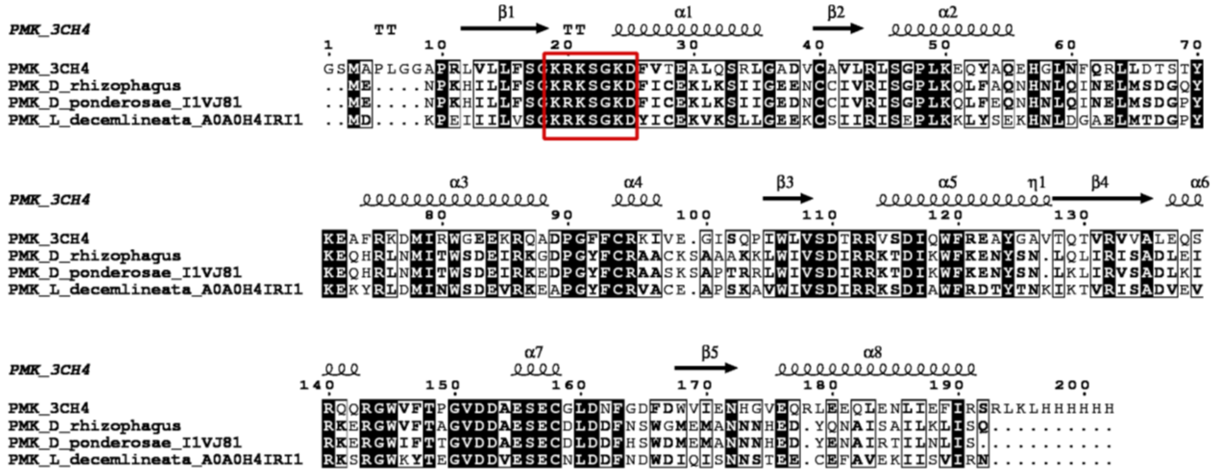

Figure S5. Multiple sequence alignment and assignment of secondary structure elements to phosphomevalonate kinase (PMK) by ESPript 3.0, according to the *Homo sapiens* protein structure (PDB: 3CH4). The multiple alignments included genus *Dendroctonus* and other coleopteran sequences. Conserved motif (red box) was manually tagged according to previous studies [114,115].

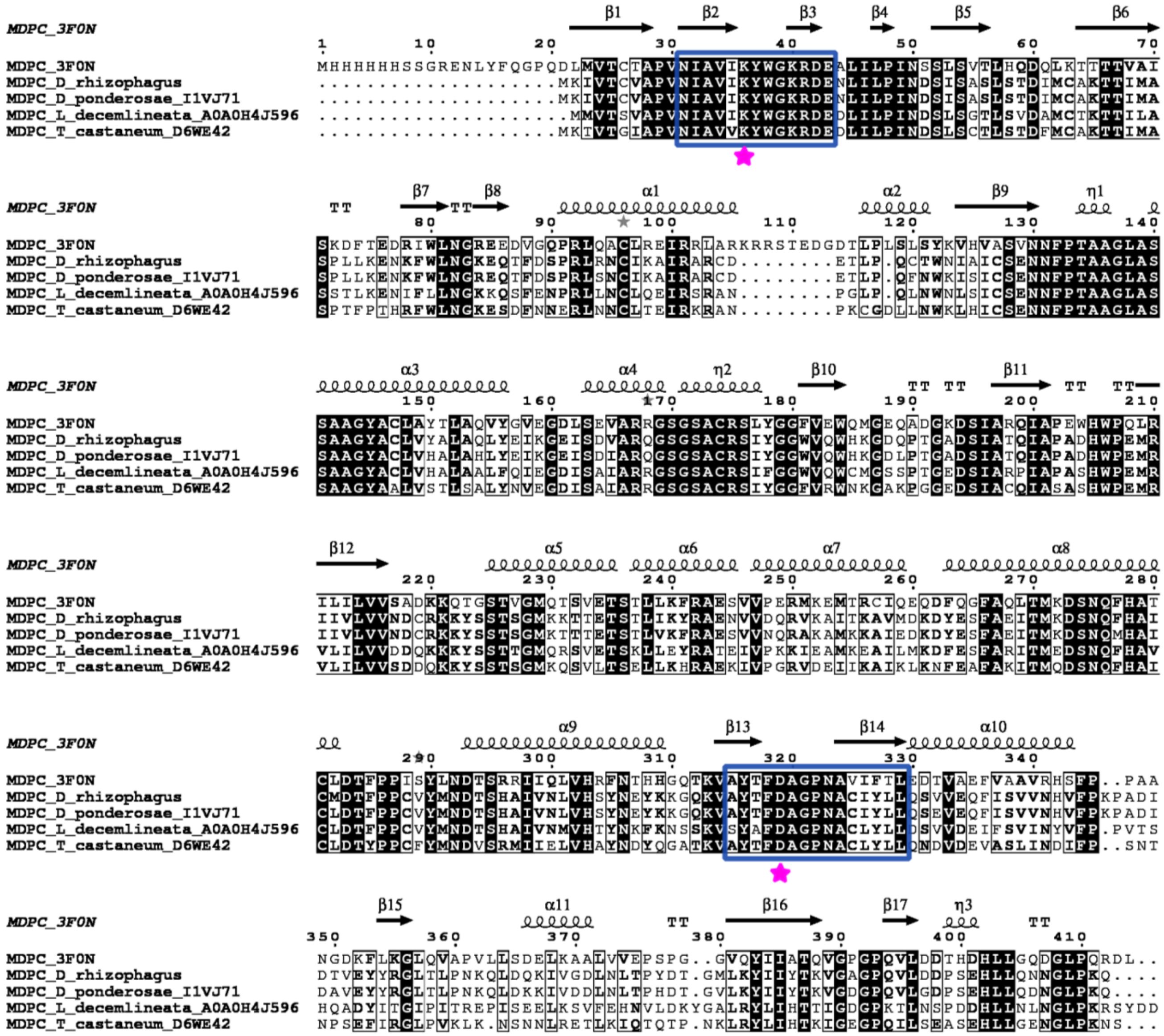

Figure S6. Multiple sequence alignment and assignment of secondary structure elements to diphosphomevalonate decarboxylase (MDPC) by ESPript 3.0, according to the *Mus musculus* protein structure (PDB: 3F0N). The multiple alignments included genus *Dendroctonus* and other coleopteran sequences. Conserved motifs (blue boxes) and amino acids involved in the catalytic activity (⋆) were manually tagged according to previous studies [116,117].

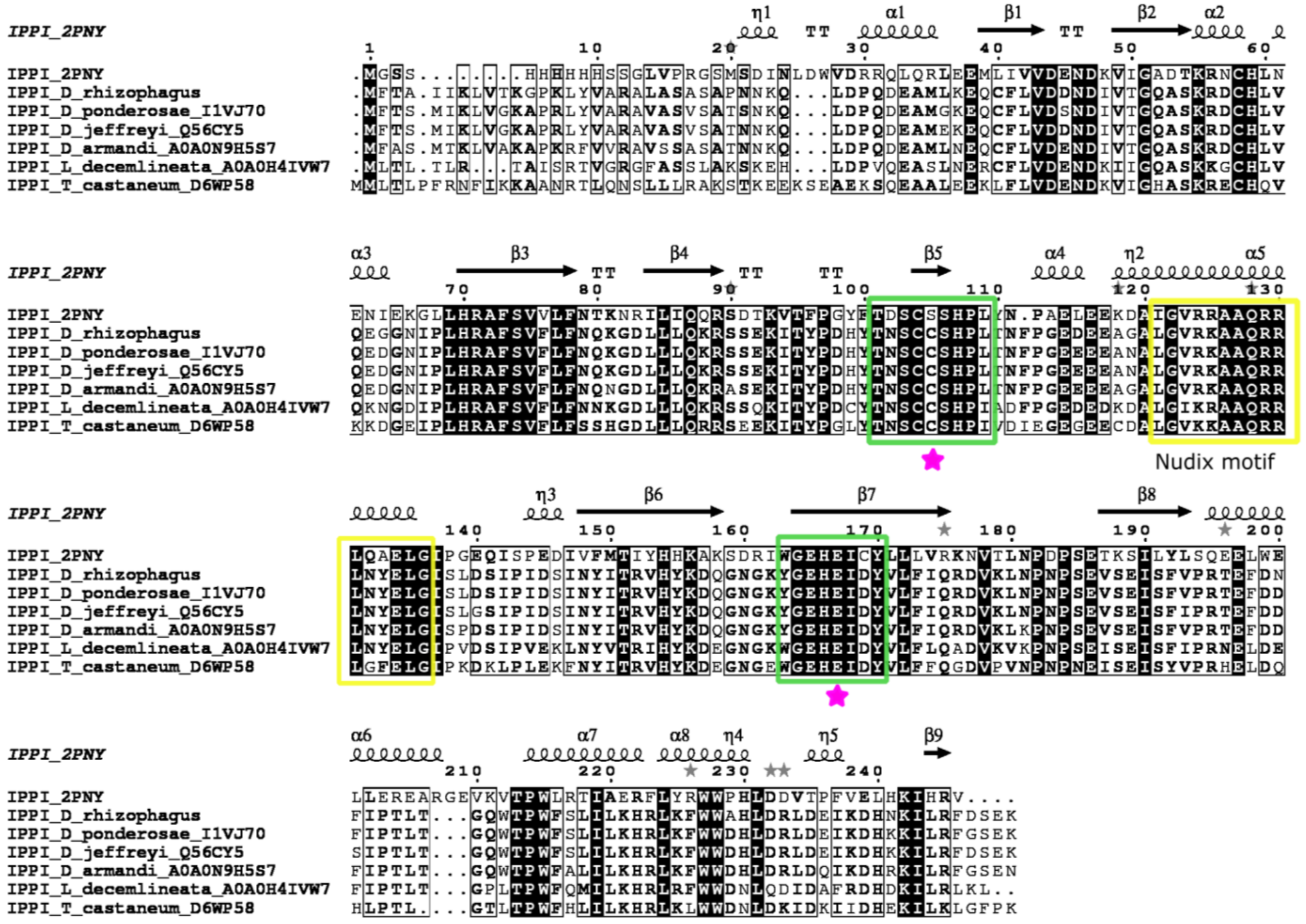

Figure S7. Multiple sequence alignment and assignment of secondary structure elements to isopentenyl diphosphate isomerase (IPPI) by ESPript 3.0, according to the *Homo sapiens* protein structure (PDB: 2PNY). The multiple alignments included genus *Dendroctonus* and other coleopteran sequences. Conserved motifs (green and yellow boxes) and amino acids involved in the catalytic activity (★) were manually tagged according to previous studies [118,119] and ExPASy-PROSITE server (http://prosite.expasy.org/).

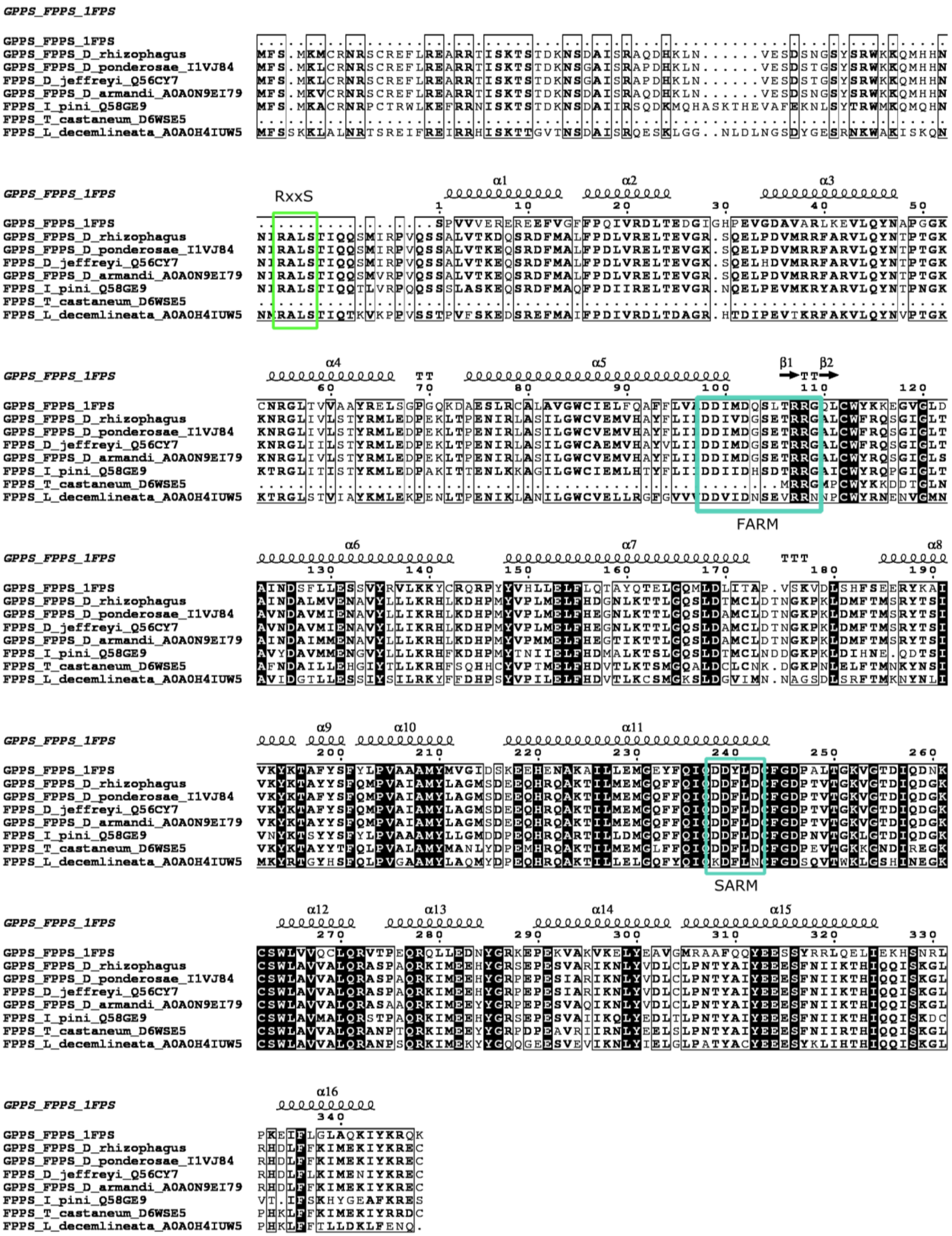

Figure S8. Multiple sequence alignment and assignment of secondary structure elements to geranyl diphosphate synthase-farnesyl diphosphate synthase (GPPS-FPPS) by ESPript 3.0, according to the *Gallus gallus* protein structure (PDB: 1FPS). The multiple alignments included genus *Dendroctonus* and other coleopteran sequences. First and Second Aspartate Rich Motifs (FARM/SARM) and putative mitochondrial cleavage site (RxxS) were manually tagged according to previous studies [33,35] and ExPASy-PROSITE server (http://prosite.expasy.org/).

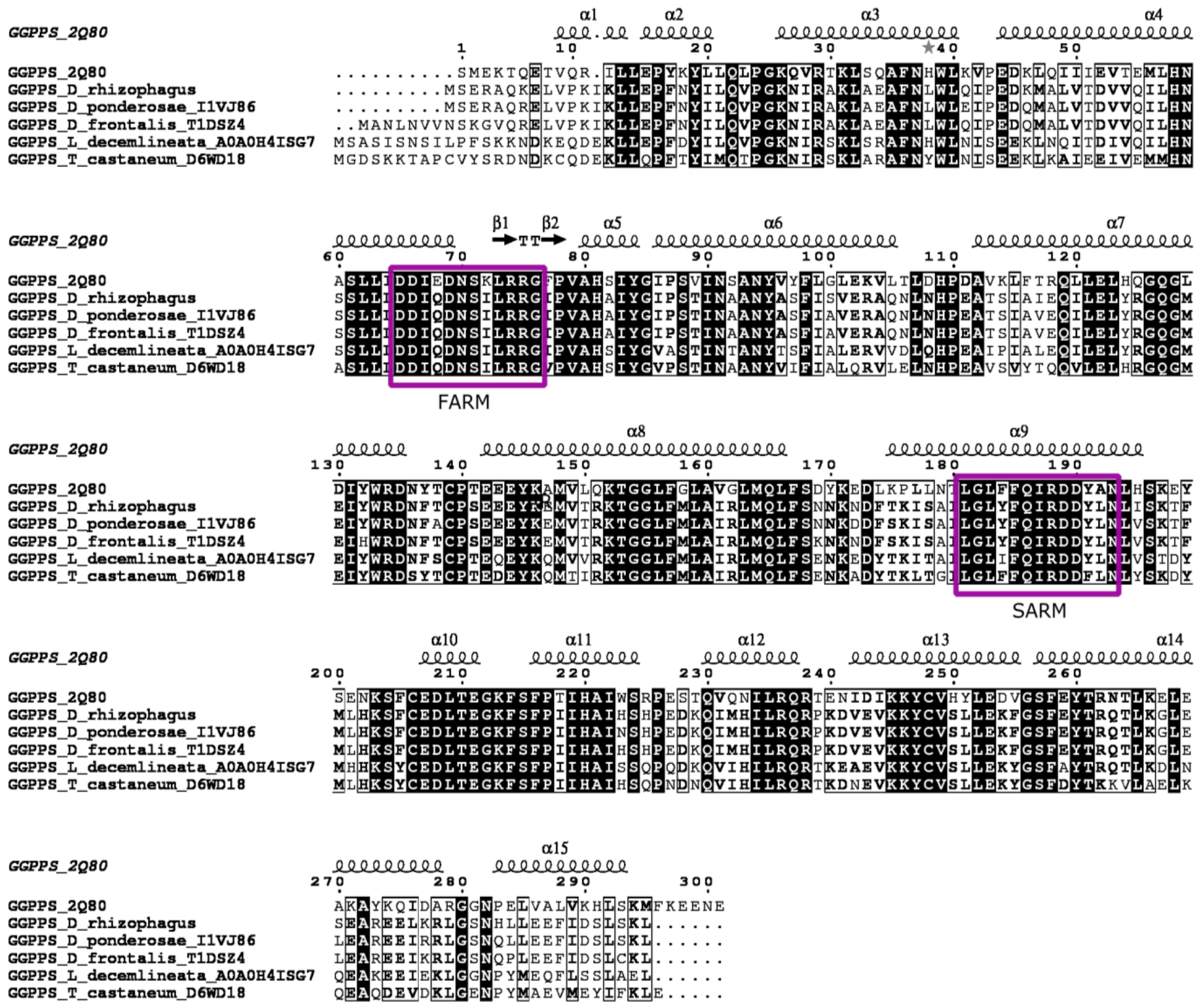

Figure S9. Multiple sequence alignment and assignment of secondary structure elements to geranylgeranyl diphosphate synthase (GGPPS) by ESPript 3.0, according to the *Homo sapiens* protein structure (PDB: 2Q80). The multiple alignments included genus *Dendroctonus* and other coleopteran sequences. First and Second Aspartate Rich Motifs (FARM/SARM) were manually tagged according to previous studies [120–122] and ExPASy-PROSITE server (http://prosite.expasy.org/).
