# Supplementary material for: The Differential Expression of Mevalonate Pathway Genes in the Gut of the Bark Beetle Dendroctonus rhizophagus (Curculionidae: Scolytinae) Is Unrelated to the de Novo Synthesis of Terpenoid Pheromones
Source: Int J Mol Sci. 2019 Aug 17;20(16):4011. doi: 10.3390/ijms20164011 (PMC6721070; doi:10.3390/ijms20164011)
Supplement: Supplementary file 1 [file ijms-20-04011-s001.zip › Supplementary Tables IJMS.pdf]

Table S1. Three-way ANOVA results from the comparison of relative expression levels of feeding treatment in *D. rhizophagus*.

		AACT		*HMGS*		*HMGR*		*MK*		*PMK*		*MDPC*		*IPPI*		*GPPS/FPPS*		*GGPPS*	
	df	*F*	*P*	*F*	*P*	*F*	*P*	*F*	*P*	*F*	*P*	*F*	*P*	*F*	*P*	*F*	*P*	*F*	*P*
Sex (S)	1	18.71	**<0.001**	71.52	**<0.001**	26.08	**<0.001**	31.07	**<0.001**	175.83	**<0.001**	27.39	**<0.001**	42.40	**<0.001**	7.71	**0.008**	0.20	0.65
Condition (C)	1	0.59	0.45	2.18	0.15	1.90	0.17	26.56	**<0.001**	2.31	0.14	23.82	**<0.001**	12.49	**0.001**	32.33	**<0.001**	9.96	**0.003**
Time (t)	1	18.64	**<0.001**	1.07	0.31	0.042	0.84	8.96	**0.005**	18.73	**<0.001**	1.74	0.19	1.87	0.18	5.74	**0.021**	2.08	0.16
S*C	1	0.93	0.34	0.95	0.33	3.29	0.077	0.72	0.400	12.04	**0.001**	4.86	**0.033**	7.47	**0.009**	1.11	0.29	1.53	0.22
S*t	1	0.003	0.96	2.82	0.10	0.32	0.57	3.15	0.084	13.78	**<0.001**	9.18	**0.004**	3.95	0.054	4.77	**0.035**	5.31	**0.026**
C*t	1	0.93	0.34	0.69	0.41	0.76	0.39	3.24	0.079	11.51	**0.002**	11.62	**0.002**	0.19	0.66	2.40	0.13	5.10	**0.029**
S*C*t	1	0.006	0.94	1.77	0.19	0.13	0.72	0.85	0.36	17.72	**<0.001**	23.11	**<0.001**	2.20	0.14	7.98	**0.007**	1.22	0.28

Bold fonts indicate significant difference.

Table S2. Two-way ANOVA results from the comparison of relative expression levels of JH III treatment in *D. rhizophagus*.

		AACT		*HMGS*		*HMGR*		*MK*		*PMK*		*MDPC*		*IPPI*		*GPPS/FPPS*		*GGPPS*	
	df	*F*	*P*	*F*	*P*	*F*	*P*	*F*	*P*	*F*	*P*	*F*	*P*	*F*	*P*	*F*	*P*	*F*	*P*
Sex (S)	1	0.76	0.39	1.09	0.31	17.73	**<0.001**	67.89	**<0.001**	2.15	0.16	5.56	**0.029**	0.30	0.59	4.69	**0.043**	0.81	0.38
Time (t)	1	0.23	0.64	8.36	**0.009**	17.77	**<0.001**	5.17	**0.034**	88.42	**<0.001**	3.33	0.083	0.34	0.57	0.66	0.42	7.09	**0.015**
S*t	1	9.29	**0.006**	11.21	**0.003**	22.51	**<0.001**	98.87	**<0.001**	0.049	0.83	2.95	0.10	4.62	**0.044**	9.58	**0.006**	4.13	0.056

Bold fonts indicate significant difference.

Table S3. Tukey's test comparisons for JH III treatment in *D. rhizophagus*.

AACT

Comparisons for factor	Comparison	Diff of Means	p	q	P	P<0.050
Sex	Males vs. Females	0.285	2	1.229	0.395	No
Time	8.000 vs. 24.000	0.157	2	0.676	0.638	No
Time within Males	8.000 vs. 24.000	1.157	2	3.527	0.022	**Yes**
Time within Females	8.000 vs. 24.000	0.844	2	2.571	0.084	No
Sex within 8	Males vs. Females	0.715	2	2.18	0.139	No
Sex within 24	Males vs. Females	1.285	2	3.918	0.012	**Yes**

HMGS

Comparisons for factor	Comparison	Diff of Means	p	q	P	P<0.050
Sex	Males vs. Females	0.533	2	1.477	0.309	No
Time	8.000 vs. 24.000	1.476	2	4.089	0.009	**Yes**
Time within Males	8.000 vs. 24.000	3.185	2	6.239	<0.001	**Yes**
Time within Females	8.000 vs. 24.000	0.233	2	0.457	0.75	No
Sex within 8	Males vs. Females	1.176	2	2.303	0.119	No
Sex within 24	Males vs. Females	2.243	2	4.392	0.006	**Yes**

HMGR

Comparisons for factor	Comparison	Diff of Means	p	q	P	P<0.050
Sex	Males vs. Females	0.461	2	5.956	<0.001	**Yes**
Time	8.000 vs. 24.000	0.461	2	5.962	<0.001	**Yes**
Time within Males	8.000 vs. 24.000	0.98	2	8.96	<0.001	**Yes**
Time within Females	8.000 vs. 24.000	0.0579	2	0.529	0.712	No
Sex within 8	Males vs. Females	0.98	2	8.956	<0.001	**Yes**
Sex within 24	Males vs. Females	0.0584	2	0.533	0.71	No

MK

Comparisons for factor	Comparison	Diff of Means	p	q	P	P<0.050
Sex	Males vs. Females	0.471	2	11.653	<0.001	**Yes**
Time	8.000 vs. 24.000	0.13	2	3.216	0.034	**Yes**
Time within Males	8.000 vs. 24.000	0.439	2	7.669	<0.001	**Yes**
Time within Females	8.000 vs. 24.000	0.699	2	12.218	<0.001	**Yes**
Sex within 8	Males vs. Females	0.0975	2	1.704	0.243	No
Sex within 24	Males vs. Females	1.04	2	18.183	<0.001	**Yes**

PMK

Comparisons for factor	Comparison	Diff of Means	p	q	P	P<0.050
Sex	Males vs. Females	0.0878	2	2.072	0.159	No
Time	8.000 vs. 24.000	0.564	2	13.298	<0.001	**Yes**
Time within Males	8.000 vs. 24.000	0.55	2	9.181	<0.001	**Yes**
Time within Females	8.000 vs. 24.000	0.577	2	9.626	<0.001	**Yes**
Sex within 8	Males vs. Females	0.101	2	1.688	0.247	No
Sex within 24	Males vs. Females	0.0745	2	1.243	0.39	No

MDPC

Comparisons for factor	Comparison	Diff of Means	p	q	P	P<0.050
Sex	Males vs. Females	2.57	2	3.336	0.029	**Yes**
Time	8.000 vs. 24.000	1.99	2	2.582	0.083	No
Time within Males	8.000 vs. 24.000	0.118	2	0.108	0.94	No
Time within Females	8.000 vs. 24.000	3.862	2	3.544	0.021	**Yes**
Sex within 8	Males vs. Females	0.698	2	0.641	0.655	No
Sex within 24	Males vs. Females	4.442	2	4.076	0.009	**Yes**

IPPI

Comparisons for factor	Comparison	Diff of Means	p	q	P	P<0.050
Sex	Males vs. Females	0.359	2	0.775	0.59	No
Time	8.000 vs. 24.000	0.38	2	0.819	0.569	No
Time within Males	8.000 vs. 24.000	1.029	2	1.571	0.28	No
Time within Females	8.000 vs. 24.000	1.788	2	2.73	0.068	No
Sex within 8	Males vs. Females	1.05	2	1.602	0.271	No
Sex within 24	Males vs. Females	1.768	2	2.698	0.071	No

GPPS/FPPS

Comparisons for factor	Comparison	Diff of Means	p	q	P	P<0.050
Sex	Males vs. Females	0.116	2	3.064	0.043	**Yes**
Time	8.000 vs. 24.000	0.0437	2	1.151	0.425	No
Time within Males	8.000 vs. 24.000	0.122	2	2.28	0.123	No
Time within Females	8.000 vs. 24.000	0.21	2	3.909	0.012	**Yes**
Sex within 8	Males vs. Females	0.0498	2	0.928	0.519	No
Sex within 24	Males vs. Females	0.282	2	5.261	0.002	**Yes**

GGPPS

Comparisons for factor	Comparison	Diff of Means	p	q	P	P<0.050
Sex	Males vs. Females	0.528	2	1.276	0.378	No
Time	8.000 vs. 24.000	1.558	2	3.765	0.015	**Yes**
Time within Males	8.000 vs. 24.000	2.746	2	4.695	0.004	**Yes**
Time within Females	8.000 vs. 24.000	0.369	2	0.63	0.661	No
Sex within 8	Males vs. Females	1.716	2	2.934	0.051	No
Sex within 24	Males vs. Females	0.661	2	1.13	0.434	No

Table S4. Tukey's test comparisons for feeding treatment in *D. rhizophagus*.

AACT

Comparisons for factor	Comparison	Diff of Means	p	q	P	P<0.050
Sex	Males vs. Females	1.826	2	6.118	<0.001	**Yes**
Condition	Paired vs. Solitary	0.325	2	1.087	0.447	No
Time	8.000 vs. 24.000	1.822	2	6.105	<0.001	**Yes**
Condition within Males	Paired vs. Solitary	0.732	2	1.733	0.228	No
Condition within Females	Paired vs. Solitary	0.0826	2	0.196	0.891	No
Sex within Solitary	Males vs. Females	1.419	2	3.362	0.022	**Yes**
Sex within Paired	Males vs. Females	2.233	2	5.29	<0.001	**Yes**
Time within Males	8.000 vs. 24.000	1.799	2	4.262	0.005	**Yes**
Time within Females	8.000 vs. 24.000	1.846	2	4.372	0.004	**Yes**
Sex within 8	Males vs. Females	1.849	2	4.381	0.004	**Yes**
Sex within 24	Males vs. Females	1.803	2	4.271	0.005	**Yes**
Time within Solitary	8.000 vs. 24.000	1.414	2	3.35	0.023	**Yes**
Time within Paired	8.000 vs. 24.000	2.23	2	5.284	<0.001	**Yes**
Condition within 8	Paired vs. Solitary	0.0836	2	0.198	0.889	No
Condition within 24	Paired vs. Solitary	0.733	2	1.736	0.227	No

HMGS

Comparisons for factor	Comparison	Diff of Means	p	q	P	P<0.050
Sex	Males vs. Females	5.604	2	11.96	<0.001	**Yes**
Condition	Paired vs. Solitary	0.978	2	2.088	0.148	No
Time	8.000 vs. 24.000	0.686	2	1.464	0.307	No
Condition within Males	Paired vs. Solitary	1.626	2	2.453	0.091	No
Condition within Females	Paired vs. Solitary	0.331	2	0.5	0.726	No
Sex within Solitary	Males vs. Females	4.957	2	7.48	<0.001	**Yes**
Sex within Paired	Males vs. Females	6.251	2	9.434	<0.001	**Yes**
Time within Males	8.000 vs. 24.000	1.799	2	2.715	0.062	No
Time within Females	8.000 vs. 24.000	0.428	2	0.645	0.651	No
Sex within 8	Males vs. Females	6.718	2	10.137	<0.001	**Yes**
Sex within 24	Males vs. Females	4.491	2	6.777	<0.001	**Yes**
Time within Solitary	8.000 vs. 24.000	0.132	2	0.199	0.889	No
Time within Paired	8.000 vs. 24.000	1.24	2	1.871	0.193	No
Condition within 8	Paired vs. Solitary	1.532	2	2.312	0.11	No
Condition within 24	Paired vs. Solitary	0.424	2	0.64	0.653	No

HMGR

Comparisons for factor	Comparison	Diff of Means	p	q	P	P<0.050
Sex	Males vs. Females	4.029	2	7.223	<0.001	**Yes**
Condition	Paired vs. Solitary	1.089	2	1.952	0.175	No
Time	8.000 vs. 24.000	0.162	2	0.291	0.838	No
Condition within Males	Paired vs. Solitary	2.519	2	3.194	0.03	**Yes**
Condition within Females	Paired vs. Solitary	0.342	2	0.433	0.761	No
Sex within Solitary	Males vs. Females	2.598	2	3.294	0.025	**Yes**
Sex within Paired	Males vs. Females	5.459	2	6.921	<0.001	**Yes**
Time within Males	8.000 vs. 24.000	0.608	2	0.771	0.589	No
Time within Females	8.000 vs. 24.000	0.284	2	0.36	0.801	No
Sex within 8	Males vs. Females	4.475	2	5.673	<0.001	**Yes**
Sex within 24	Males vs. Females	3.583	2	4.542	0.003	**Yes**
Time within Solitary	8.000 vs. 24.000	0.849	2	1.076	0.451	No
Time within Paired	8.000 vs. 24.000	0.525	2	0.665	0.641	No
Condition within 8	Paired vs. Solitary	0.402	2	0.51	0.721	No
Condition within 24	Paired vs. Solitary	1.776	2	2.251	0.119	No

MK

Comparisons for factor	Comparison	Diff of Means	p	q	P	P<0.050
Sex	Males vs. Females	0.8	2	7.883	<0.001	**Yes**
Condition	Paired vs. Solitary	0.739	2	7.288	<0.001	**Yes**
Time	8.000 vs. 24.000	0.429	2	4.233	0.005	**Yes**
Condition within Males	Paired vs. Solitary	0.861	2	6.004	<0.001	**Yes**
Condition within Females	Paired vs. Solitary	0.617	2	4.303	0.004	**Yes**
Sex within Solitary	Males vs. Females	0.678	2	4.724	0.002	**Yes**
Sex within Paired	Males vs. Females	0.922	2	6.425	<0.001	**Yes**
Time within Males	8.000 vs. 24.000	0.684	2	4.767	0.002	**Yes**
Time within Females	8.000 vs. 24.000	0.175	2	1.219	0.394	No
Sex within 8	Males vs. Females	1.054	2	7.349	<0.001	**Yes**
Sex within 24	Males vs. Females	0.545	2	3.8	0.011	**Yes**
Time within Solitary	8.000 vs. 24.000	0.171	2	1.193	0.404	No
Time within Paired	8.000 vs. 24.000	0.688	2	4.794	0.002	**Yes**
Condition within 8	Paired vs. Solitary	0.998	2	6.954	<0.001	**Yes**
Condition within 24	Paired vs. Solitary	0.481	2	3.353	0.023	**Yes**

PMK

Comparisons for factor	Comparison	Diff of Means	p	q	P	P<0.050
Sex	Males vs. Females	4.16	2	18.75	<0.001	**Yes**
Condition	Solitary vs. Paired	0.477	2	2.15	0.136	No
Time	8.000 vs. 24.000	1.358	2	6.12	<0.001	**Yes**
Sex within Solitary-8h	Males vs. Females	7.733	2	17.43	<0.001	**Yes**
Sex within Paired-8h	Males vs. Females	2.915	2	6.57	<0.001	**Yes**
Condition within Males-8	Solitary vs. Paired	3.951	2	8.90	<0.001	**Yes**
Sex within 24	Males vs. Females	2.995	2	9.55	<0.001	**Yes**

MDPC

Comparisons for factor	Comparison	Diff of Means	p	q	P	P<0.050
Sex	Males vs. Females	0.837	2	7.401	<0.001	**Yes**
Condition	Solitary vs. Paired	0.781	2	6.903	<0.001	**Yes**
Time	8.000 vs. 24.000	0.211	2	1.866	0.195	NO
Sex within Paired-8h	Males vs. Females	2.444	2	10.8	<0.001	**Yes**
Condition within Males-8	Solitary vs. Paired	2.448	2	10.82	<0.001	**Yes**

IPPI

Comparisons for factor	Comparison	Diff of Means	p	q	P	P<0.050
Sex	Males vs. Females	1.575	2	9.209	<0.001	**Yes**
Condition	Paired vs. Solitary	0.855	2	4.999	0.001	**Yes**
Time	8.000 vs. 24.000	0.331	2	1.932	0.18	No
Condition within Males	Paired vs. Solitary	1.517	2	6.269	<0.001	**Yes**
Condition within Females	Paired vs. Solitary	0.194	2	0.801	0.574	No
Sex within Solitary	Males vs. Females	0.914	2	3.778	0.011	**Yes**
Sex within Paired	Males vs. Females	2.237	2	9.246	<0.001	**Yes**
Time within Males	8.000 vs. 24.000	0.812	2	3.354	0.023	**Yes**
Time within Females	8.000 vs. 24.000	0.15	2	0.622	0.663	No
Sex within 8	Males vs. Females	2.056	2	8.5	<0.001	**Yes**
Sex within 24	Males vs. Females	1.094	2	4.524	0.003	**Yes**
Time within Solitary	8.000 vs. 24.000	0.223	2	0.921	0.519	No
Time within Paired	8.000 vs. 24.000	0.438	2	1.811	0.208	No
Condition within 8	Paired vs. Solitary	0.963	2	3.98	0.008	**Yes**
Condition within 24	Paired vs. Solitary	0.748	2	3.09	0.035	**Yes**

GPPS/FPPS

Comparisons for factor	Comparison	Diff of Means	p	q	P	P<0.050
Sex	Males vs. Females	0.246	2	3.926	0.008	**Yes**
Condition	Solitary vs. Paired	0.504	2	8.041	<0.001	**Yes**
Time	8.000 vs. 24.000	0.212	2	3.389	0.021	**Yes**
Sex within Paired-8h	Males vs. Females	0.783	2	6.251	<0.001	**Yes**
Condition within Males-8	Solitary vs. Paired	0.71	2	5.668	<0.001	**Yes**
Condition within 24 h	Solitary vs. Paired	0.641	2	7.236	<0.001	**Yes**

GGPPS

Comparisons for factor	Comparison	Diff of Means	p	q	P	P<0.050
Sex	Males vs. Females	0.103	2	0.636	0.655	No
Condition	Paired vs. Solitary	0.72	2	4.463	0.003	**Yes**
Time	8.000 vs. 24.000	0.329	2	2.039	0.157	No
Condition within Males	Paired vs. Solitary	0.438	2	1.919	0.183	No
Condition within Females	Paired vs. Solitary	1.002	2	4.393	0.004	**Yes**
Sex within Solitary	Males vs. Females	0.385	2	1.687	0.24	No
Sex within Paired	Males vs. Females	0.18	2	0.787	0.581	No
Time within Males	8.000 vs. 24.000	0.197	2	0.864	0.545	No
Time within Females	8.000 vs. 24.000	0.855	2	3.747	0.012	**Yes**
Sex within 8	Males vs. Females	0.423	2	1.855	0.197	No
Sex within 24	Males vs. Females	0.629	2	2.755	0.059	No
Time within Solitary	8.000 vs. 24.000	0.187	2	0.818	0.567	No
Time within Paired	8.000 vs. 24.000	0.844	2	3.701	0.013	**Yes**
Condition within 8	Paired vs. Solitary	1.236	2	5.415	<0.001	**Yes**
Condition within 24	Paired vs. Solitary	0.205	2	0.897	0.53	No

Table S5. Primers designed for cloning and RT-qPCR analysis of MVA pathway genes in *D. rhizophagus*.

Gene		Primers sequences (5′ → 3′)	Location of amplicon & Amplicon length (bp)	Application	Annealing temperature (°C) & Extension time (s)
AACT	F<br>R<br>P	GGC GAA TGC CTC CAC TTT G<br>GCC GCT CTT GTG GTT AAA ATC AG<br>CCG CAG CTC CGT CAT T	834-893<br>60 bp	RT-qPCR<br>(Assay ID. AI6RPRP)	60 °C / 60 s
HMGS	F<br>R<br>P	TGT TAC CAG CTT TAC TGC CAG AAG<br>CAA ACG ACT CAA TAC TCA CAG GCT<br>CTG CAA CAG CTT TTC C	667-733<br>67 bp	RT-qPCR<br>(Assay ID. AI70NXX)	″
HMGR	F<br>R<br>P	CCA AGC TCC TTG AAA ACT TAC CTT TT<br>CCA CTG GTA TTG GCA TGT ATC CA<br>CAC GCA CCC ATG ACC AA	1382-1480<br>99 bp	RT-qPCR<br>(Assay ID. AI89L35)	″
MK	F<br>R<br>P	GGT GTG TAC TTT TGG CAA TAT GGT<br>GCA CTT TGC TAT TTA CGA GCA GTA A<br>CCG TCG TTC ATC TCT C	762-862<br>101 bp	RT-qPCR<br>(Assay ID. AIAA09Y)	″
PMK	F<br>R<br>P	ACT TGG AGT GAT GAA ATC AGA AAG GG<br>GCC GCT GAT TTG CAA GCT<br>CCG GGC TAC TTT TGC A	220-287<br>68 bp	RT-qPCR<br>(Assay ID. AIBJZF6)	″
MDPC	F<br>R<br>P	GAC AGG GTT CTG GAA GTG CAT<br>TCA CCC TTG TGC CAT TGC A<br>CCA ACC ACC ATA AAT AC	413-476<br>64 bp	RT-qPCR<br>(Assay ID. AICSXME)	″
IPPI	F<br>R<br>P	AGA CAG CAT TCC CAT TGA CAG TAT TAA TT<br>CGA TCT CAT GCT CGC CAT ATT TG<br>CCG TTC CCT TGG TCT TT	432-520<br>89 bp	RT-qPCR<br>(Assay ID. AID1VSM)	″
GPPS/FPPS	F<br>R<br>P	GCC AAA ACG ATC CTG ATG GAA ATG<br>GCC GAA GCA ATC CAG AAA ATC G<br>TTG AAT CTG GAA AAA CTG	910-978<br>69 bp	RT-qPCR<br>(Assay ID. AIFATYU)	″
GGPPS	F<br>R<br>P	CAC CTG TCC CTC TGA GGA AGA<br>CTG ATG GCC AGC ATG AAC AAC<br>CCG CCC GTT TTT CGA G	417-491<br>75 bp	RT-qPCR<br>(Assay ID. AIGJR42)	″
CYP4G55v1	F<br>R<br>P	GACTATGCAATGGCTGTGATGAAG<br>GATGTAGTCGGAGTAAAGCCAGATT<br>CTGTGCGACATTCTC	688-768<br>81 bp	RT-qPCR<br>(Assay ID. AIS065U)<br>[40]	″
AACT	F<br>R	AAC ATT TAG ACT CAG CTA TTG GG<br>ACA ACT ACT CGA ACA TGA ACG C	1340 bp	cDNA cloning	61°C / 80 s
HMGS	F<br>R	ATG AGT GCC TGG CCT GAA GAT GTG<br>CTA TGA GTG GCC GTT GGT GAT CAT	1374 bp	cDNA cloning	66 °C / 85 s
HMGR	F1<br>R1	GGA ATA ACT TGG ATA TTC TCA TCT G<br>GTG TCT CTA CCA GTG TAT CTA AAG	500 bp	cDNA cloning	60°C / 35 s
	F2<br>R2	GCT CAA TTC GCT CTT AAT GGG G<br>AGC TCT ACA TCC TCT ATT AGT GC	1155 bp		58 °C / 75 s
	F3<br>R3	GCT TCT GGA TGG TCG TGA GTT GTA<br>TAA GGT AGT GGA TCG GTT GTG C	991 bp		60 °C / 60 s
	F4<br>R4	ATA GGT GGA TTC AAC GCT CAT GCT G<br>GCT ACA TTT TTA AGC ATT GTC TTG GC	400 bp		60 °C / 35 s
MK	F<br>R	GCA AAC TTT AAC TAC CAA GCA GTG<br>TTA ATC AAC TCT AAC CCC TGG AC	1350 bp	cDNA cloning	64 °C / 85 s
PMK	F<br>R	GCG AAA TTA AGA ATC AGT AAT ACC T<br>AAA TCG TCT AGG TCG CAT TCG	511 bp	cDNA cloning	62 °C / 40 s
	F<br>R	CTG GTA AAG ACT TCA TTT GTG AG<br>CTA TAT TGT GCC GCC ACC AAA GG	550 bp		62 °C / 40 s
MDPC	F<br>R	ATT CCT AAA GTA GCG AGT TGT G<br>GCA CCG TTC ATC AGT TTG ACT TCA	1200 bp	cDNA cloning	61 °C / 75 s
IPPI	F<br>R	GTT TAT CAG TGG ACC ATT GTG CTG<br>GTT CTT AAA CAT AGA TGA CTA GCA G	1100 bp	cDNA cloning	59 °C / 65 s
GPPS/FPPS	F<br>R	ATG TTT TCG ATG AAA ATG TGC CGC<br>TCC CTA AGG AGC TTC TAA CAC TCG	1290 bp	cDNA cloning	63 °C / 80 s
GGPPS	F<br>R	ACC ACA AAA CCT TTC CCA AAT T<br>TTC CTG GAA TTA TTA CAA GAA CTT	1000 bp	cDNA cloning	56 °C / 60 s

*F: Forward, R: Reverse, P: TaqMan™ Probe.

Table S6. Minimum Information for Publication of Quantitative Real-Time PCR Experiments (MIQE) checklist.

**Item to check**	**Details**
Experimental design	
Definition of experimental and control groups	yes, included in the manuscript
Number within each group	yes, included in the manuscript
Assay carried out by the core or investigator's laboratory?	yes, all experiments were performed in our laboratory
Acknowledgment of authors' contributions	yes, included in the manuscript
Sample	
Description	yes, included in the manuscript
Volume/mass of sample processed	yes, included in the manuscript
Microdissection or macrodissection	yes, included in the manuscript
Processing procedure	yes, included in the manuscript
If frozen, how and how quickly?	this does not apply
If fixed, with what and how quickly?	this does not apply
Sample storage conditions and duration (especially for FFPEb samples)	this does not apply
Nucleic acid extraction	
Procedure and/or instrumentation	yes, we followed the manufacturer's protocol without modification
Name of kit and details of any modifications	yes, included in the manuscript. We used a commercial RiboPure™ kit (Ambion by Life Technologies, Cat. No. AM1924)
Source of additional reagents used	this does not apply, as we did not used any other additional reagent
Details of DNase or RNase treatment	we did not performed DNase or RNase treatment
Contamination assessment (DNA or RNA)	this was evaluated by means of A_{260}/A_{280} absorbance
Nucleic acid quantification	
Instrument and method	yes, this is included in the manuscript
Purity (A260/A280)	yes, this was evaluated
Yield	yes, we described the yield estimation in the manuscript
RNA integrity: method/instrument	yes, we described the yield estimation in the manuscript
RIN/RQI or Cq of 3′ and 5′ transcripts	this was not evaluated
Electrophoresis traces	yes, included in the manuscript
Inhibition testing (Cq dilutions, spike, or other)	this was not evaluated
Reverse transcription	
Complete reaction conditions	yes, we followed all manufacturer's protocols with no single modification
Amount of RNA and reaction volume	yes, included in the manuscript
Priming oligonucleotide (if using GSP) and concentration	this does not apply
Reverse transcriptase and concentration	the information is not provided by the manufacturer
Temperature and time	we followed manufacturer's protocol: 37°C for 60 minutes and 95°C for 5 minutes
Manufacturer of reagents and catalogue numbers	yes, included in the manuscript. We used a commercial High Capacity RNA to cDNA kit (Applied Biosystems, USA; Cat. No. 4387406)
C_qs with and without reverse transcription	this was not evaluated
Storage conditions of cDNA	yes, included in the manuscript
qPCR target information	
Gene symbol	yes, included in the manuscript
Sequence accession number	yes, included in the manuscript
Location of amplicon	yes, included in the supplementary data
Amplicon length	yes, included in the supplementary data
In silico specificity screen (BLAST, and so on)	yes, this was done by the manufacturer (Applied Biosystems)
Pseudogenes, retropseudogenes, or other homologs?	does not apply
Sequence alignment	yes, this was done by the manufacturer (Applied Biosystems)
Secondary structure analysis of amplicon	yes, this was done by the manufacturer (Applied Biosystems)
Location of each primer by exon or intron (if applicable)	does not apply
What splice variants are targeted?	does not apply
qPCR oligonucleotides	
Primer sequences	yes, included in the supplementary data
RTPrimerDB identification number	does not apply
Probe sequences	yes, included in the supplementary data
Location and identity of any modifications	does not apply
Manufacturer of oligonucleotides	yes, included in the manuscript

Purification method	the information provided by the manufacturer indicates that oligonucleotides are purified via Transgenomic Wave HPLC. The method is proprietary
qPCR protocol	
Complete reaction conditions	yes, included in the manuscript
Reaction volume and amount of cDNA/DNA	yes, included in the manuscript
Primer, (probe), Mg^{2+}, and dNTP concentrations	yes, concentration of the primers and probe are in the manuscript; however, the concentration of the master mix is not provided by the manufacturer
Polymerase identity and concentration	AmpliTaq Gold® DNA polymerase, UP; dNTPs with dUTP; ROX™ passive reference; optimized buffer components.
Buffer/kit identity and manufacturer	this is included along with the PCR master mix II (Cat No. 4427788), but the information is not provided by the manufacturer
Exact chemical composition of the buffer	this is included along with the PCR master mix II (Cat No. 4427788), but the information is not provided by the manufacturer
Additives (SYBR Green I, DMSO, and so forth)	does not apply
Manufacturer of plates/tubes and catalog number	only 48-well plates were used (MicroAmp™, Cat. No.4375816) and optical adhesive film (MicroAmp™, Cat. No. 4375928) (Applied Biosystems, USA)
Complete thermocycling parameters	yes, included in the manuscript
Reaction setup (manual/robotic)	manual
Manufacturer of qPCR instrument	yes, included in the manuscript
qPCR validation	
Evidence of optimization (from gradients)	yes, available upon request
Specificity (gel, sequence, melt, or digest)	yes, this is described in the manuscript
For SYBR Green I, Cq of the NTC	does not apply
Calibration curves with slope and *y* intercept	yes, available upon request
PCR efficiency calculated from slope	yes, available upon request
CIs for PCR efficiency or SE	yes, available upon request
r^2 of calibration curve	yes, this is described in the manuscript
Linear dynamic range	yes, they are available upon request
C_q variation at LOD	this does not apply
CIs throughout range	yes, available upon request
Evidence for LOD	this does not apply
If multiplex, efficiency and LOD of each assay	this does not apply
Data analysis	
qPCR analysis program (source, version)	yes, included in the manuscript.
Method of Cq determination	Cq is determined by the StepOne™ Software v2.3 in the geometric phase where the standard deviation between replicates is minor
Outlier identification and disposition	Outliers were automatically identified by the qPCR instrument software. Once this, they were eliminated from the analysis. However, when only one out of three group replicates were eliminated, the remaining two were subsequently used in the analysis
Results for NTCs	yes, this was not reported
Justification of number and choice of reference genes	does not apply
Description of normalization method	yes, included in the manuscript
Number and concordance of biological replicates	yes, included in the manuscript
Number and stage (reverse transcription or qPCR) of technical replicates	yes, included in the manuscript
Repeatability (intraassay variation)	yes, we estimated this as SD for the C_q variance and are available upon request
Reproducibility (interassay variation, CV)	does not apply
Power analysis	this was not evaluated
Statistical methods for results significance	yes, included in the manuscript
Software (source, version)	yes, included in the manuscript. The program used for the analysis was SigmaStat v. 3.5.
C_q or raw data submission with RDML	this was not reported
